# Platelet dysfunction in immune thrombocytopenia: Finding clinical subsets with platelet phenotypes

**DOI:** 10.1111/bjh.70179

**Published:** 2025-09-29

**Authors:** Sidra A. Ali, Sarah M. Hicks, Lucy A. Coupland, Simone A. Brysland, Vijay Bhoopalan, Yee Lin Thong, Amandeep Kaur, Robert K. Andrews, Elizabeth E. Gardiner, Philip Y.‐I. Choi

**Affiliations:** ^1^ Division of Genome Science and Cancer, John Curtin School of Medical Research Australian National University Canberra Australian Capital Territory Australia; ^2^ Ingham Institute for Applied Medical Research Liverpool New South Wales Australia; ^3^ The National Platelet Research and Referral Centre Canberra Australian Capital Territory Australia; ^4^ Haematology Department, the Canberra Hospital, Woden Canberra Australia

**Keywords:** bleeding, immune thrombocytopenia, platelet, receptor, ROTEM

## Abstract

Patients with immune thrombocytopenia (ITP) remain a challenge to diagnose, manage and predict bleeding risk. A comprehensive assessment of platelet function may aid clinical management. This study assessed platelet parameters to predict bleeding in ITP. Blood from 103 clinically annotated cases with isolated thrombocytopenia and 123 healthy donors was evaluated. In the ITP cohort, 75/110 encounters (68%) had platelet counts below 50 × 10^9^/L. Platelet surface proteins, reticulated platelets and activation were quantified using flow cytometry. Soluble receptor fragments, citrullinated histone‐DNA (CitH3‐DNA) complexes and thrombopoietin (TPO) were quantified by enzyme‐linked immunosorbent assay (ELISA). Whole blood clotting and platelet contribution to clot formation were evaluated using viscoelastography. Elevated levels of glycoprotein (GP) VI (*p* = 0.0012), Trem‐like transcript‐1 (TLT‐1) (*p* = 0.0248), platelet‐bound immunoglobulin (Ig) G (*p* < 0.0029), CitH3‐DNA complexes (*p* = 0.0022), TPO (*p* < 0.0001) and reduced platelet contribution to clot formation (*p* < 0.0001) were observed in primary ITP patients with bleeding and bruising symptoms. A multivariable analysis revealed that measuring platelet indices strengthened a predictive bleeding model over platelet count alone (78.1% vs. 70.48%). Symptomatic ITP patients have measurable platelet dysfunction and quantifiable differences on platelet surface, increased evidence of NETosis and elevated TPO levels. Identifying biomarkers for ITP outcomes can define subsets of disease with clinical relevance.

## INTRODUCTION

Immune thrombocytopenia (ITP) is a rare disease, with an estimated incidence in adults ranging from 1.6 to 3.9 cases per 100 000 per year.^1^ Insights into the pathogenic mechanisms underlying ITP and the advent of therapies, including thrombopoietin receptor agonists (TPO‐RA) and rituximab, have changed the landscape of ITP management. Still, a challenging minority of patients remain refractory to multiple lines of treatment and develop chronic forms of thrombocytopenia, repeated bleeding episodes, poor health‐related quality of life and high mortality.^2^ ITP is a broad and imprecise diagnostic classification used to describe several molecularly distinct disorders^3^ that may include varying contributions by platelet‐specific autoantibodies, loss of regulation of immune function and acquired megakaryocyte maturation abnormalities.^4^, ^5^

Platelets maintain blood volume and aid innate immune responses and wound repair.^6^ In ITP, key platelet receptors, including glycoprotein (GP) Ib‐IX‐V complex, GPV, GPVI and integrins αIIbβ3 and α2β1, are targeted by anti‐platelet autoantibodies^7^ which can mediate platelet activation via fragment crystallisable (Fc)‐dependent and ‐independent means.^8^ Major challenges in ITP include a paucity of definitive diagnostic tools and a lack of clinical acuity regarding the ideal therapeutic option for each patient. As platelets are targeted in ITP, assessing their function makes sense but is challenging as samples need to be collected and rapidly analysed, ideally using assays that are insensitive to platelet numbers.

Rapid, point‐of‐care testing of platelet function also provides significant benefit to determine bleeding risk, as shown in a pilot study of ITP cases with comparable thrombocytopenia.^9^ Non‐time‐sensitive measures of platelet activation and function are a clinical unmet need. Flow cytometry is an appealing alternative, as it is largely unaffected by the platelet count. Further, platelet binding by anti‐platelet autoantibodies, which is central to ITP pathogenesis,^1^, ^10^, ^11^, ^12^ can cause rapid and irreversible proteolytic release of receptor ectodomains such as soluble GPVI (sGPVI) and soluble triggering receptor expressed on myeloid cells‐like (Trem)‐like transcript‐1 (sTLT‐1).^13^ Measurement of sGPVI and sTLT‐1 reflects platelet activation and degranulation. Activated platelets also promote the formation of neutrophil extracellular traps (NETs), which are implicated in immune‐mediated cellular destruction and correlate with clinical severity.^14^ These biomarkers were selected for their relevance to known ITP mechanisms, including immune‐mediated platelet activation, receptor shedding and platelet–immune cell interactions.

Our objective here was to combine flow cytometric evaluation of platelet quality and function, along with viscoelastography and measurement of soluble plasma or serum proteins in blood drawn from ITP patients, to develop a platelet signature that could aid clinical diagnosis, predict clinical course and ultimately help stratify patients with ITP for appropriate therapy. With the range of emerging targetable aspects of ITP,^15^, ^16^, ^17^, ^18^ diagnostic tools to aid the logical selection of therapy become increasingly imperative.

## METHODS

### Whole blood collection and clinical annotations

The National Platelet Research and Referral Centre (NPRC) is a specialist centre for platelet evaluation within the John Curtin School of Medical Research and Canberra Health Services. The NPRC hosts a biobank of plasma samples and an annotated clinical database that documents bleeding episodes, disease duration and treatment responses (Table S1). Non‐consecutive patients were referred for NPRC biobanking with disorders of platelet number (<100 × 10^9^/L) or suspected platelet dysfunction.

Patients with WHO bleeding (≥ grade 1) or an Immune thrombocytopenia ‐ specific Bleeding Assessment Tool (ITP‐BAT) score ≥1^19^ were pooled and compared to asymptomatic patients. Patients receiving steroids and/or intravenous immunoglobulin (IVIg) were classified as undergoing first‐line therapy, while those receiving any other treatments were grouped under second‐line therapy. This included TPO‐RA, rituximab, mycophenolate mofetil, dapsone, ciclosporin, danazol, vincristine, azathioprine, oseltamivir, ianalumumab and efgartigimod or splenectomy.

### Flow cytometry analysis

Details can be found in Methods S1. Reticulated platelets were quantified using thiazole orange (TO). To quantify platelet surface proteins, trisodium citrate (TSC)‐anti‐coagulated whole blood (WB) was incubated with fluorescently conjugated antibodies against platelet‐specific proteins, and antibody‐bound platelet events were acquired (Figure S1). To assess platelet αIIbβ3 activation (Figure S2A for gating strategy), WB was stimulated with platelet agonists in the presence of Oregon‐Green‐tagged fibrinogen (OG‐Fg). FlowJo™ software (v10.8 BD Life Sciences) was used for analysis.

### Viscoelastography with rotational thromboelastometry (ROTEM)

All samples also underwent ROTEM analysis within 4 h of venepuncture. To gain an indication of the platelet contribution to clot formation, platelet A10 was calculated by subtracting FibTEM A10 from EXTEM A10. FibTEM uses cytochalasin D, an actin polymerisation inhibitor, to block platelet cytoskeletal changes, resulting in a fibrin‐dominant clot. In contrast, EXTEM reflects both platelet and fibrin contributions.

Detailed descriptions of all other experimental methods are provided in Methods S1.

### Statistical analysis

Details can be found in Methods S1.

## RESULTS

### NPRC patient cohort overview

**FIGURE 1:**
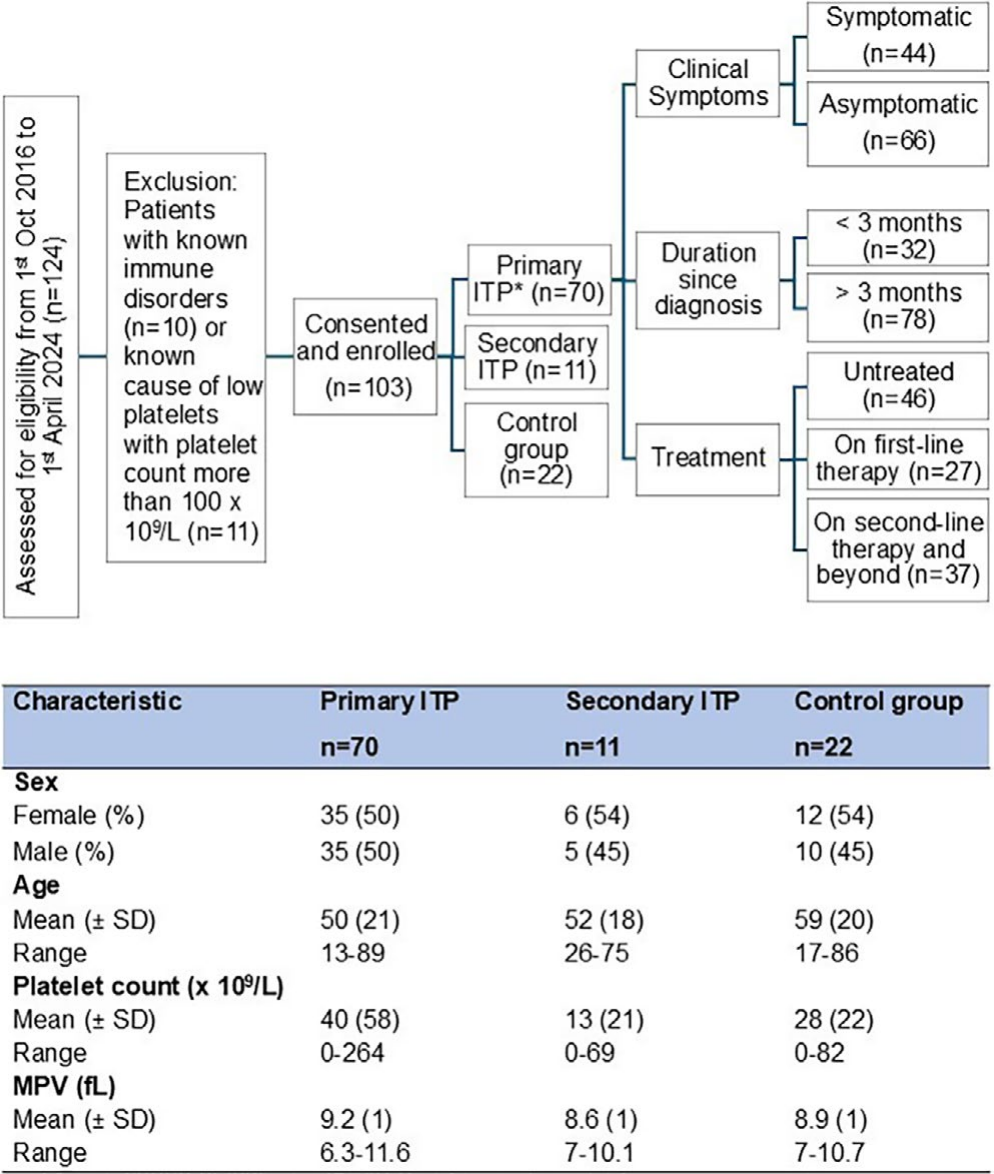
Patient recruitment flow chart and demographics. Control group = patients with platelet count below 100 × 10^9^/L due to causes other than ITP. Patients receiving steroids and/or IVIg at the time of blood collection were classified as undergoing first‐line therapy, while the remaining patients were receiving second‐line therapy and beyond. ITP, immune thrombocytopenia; MPV, mean platelet volume. *110 encounters from primary ITP patients, involving 70 individual patients, with repeated sample collections (*n* = 21) at different time points, including during remission.

The NPRC investigates platelet phenotypes in patients with thrombocytopenia using research‐based assays. Between 1 October 2016 and 1 April 2024, 103 patients were recruited, and blood samples and clinical data were collected. Cohort characteristics (Figure 1) show 68% (70/103) of patients were diagnosed with primary ITP (pITP), 10% (11/103) with secondary ITP (sITP) with a known underlying aetiology and 21% (22/103) with platelet counts below 100 × 10^9^/L due to causes other than ITP (control thrombocytopenic group; Table S2).

**FIGURE 2:**
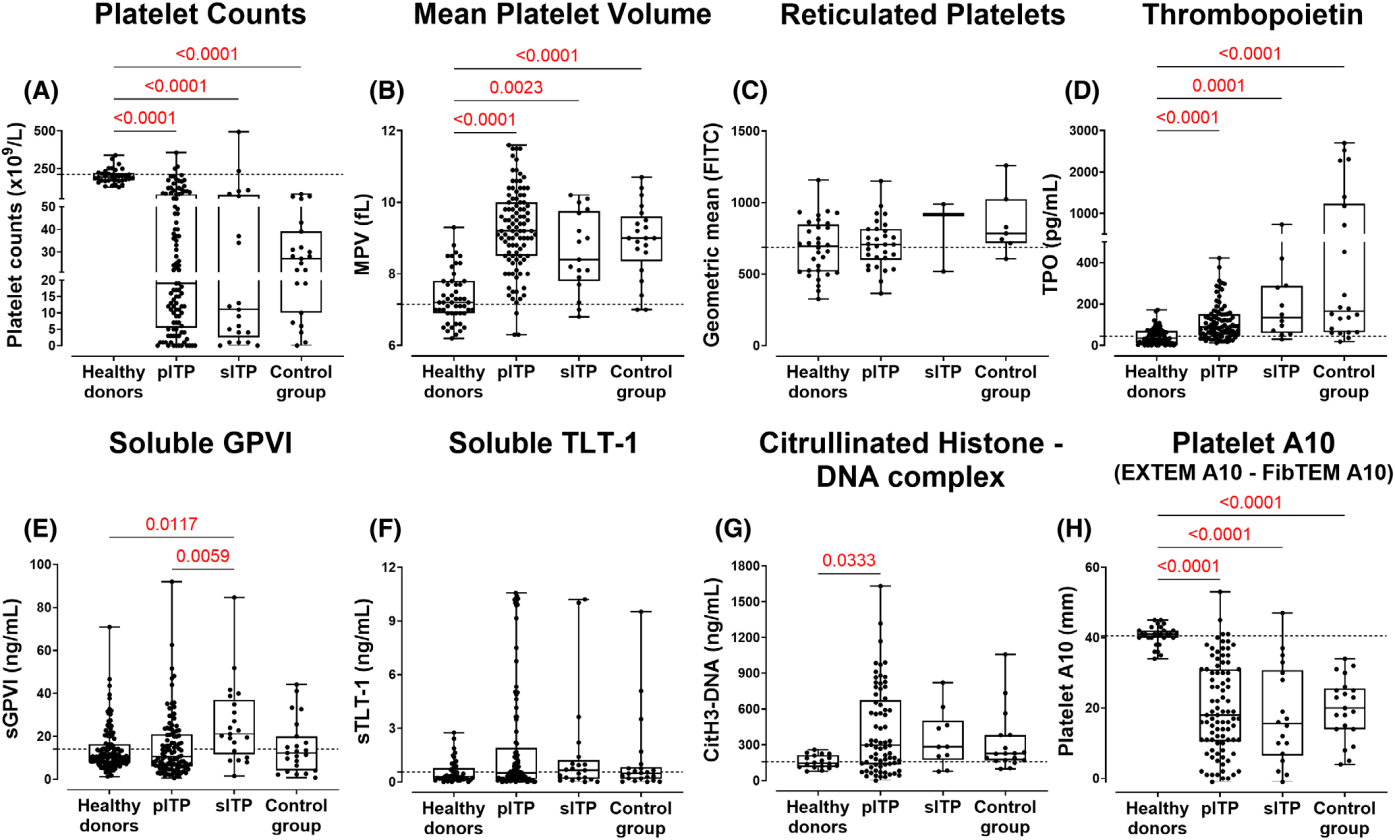
Platelet and plasma parameters in healthy donors, ITP patients and control group. (A) Platelet counts and (B) MPV were measured using an automated haematology analyser in TSC‐anti‐coagulated WB. (C) Reticulated platelets were quantified by TO staining in flow cytometry. Levels of (D) thrombopoietin (TPO). (E) Soluble (s) GPVI, (F) sTLT‐1 and (G) citrullinated histone‐DNA (CitH3‐DNA) complex were measured by ELISA. (H) Platelet A10 was calculated by subtracting the FibTEM A10 measurement from the EXTEM A10 in ROTEM in healthy donors (*n* = 18–123), pITP patients (*n* = 30–105), sITP patients (*n* = 3–12) and control groups (*n* = 7–23). Only significant *p*‐value (*p* < 0.05) highlighted in red is displayed. Data include repeat measurements from the same subjects collected at different time points. The dotted horizontal line represents the healthy donor mean measured alongside these patients. ITP, immune thrombocytopenia; MPV, mean platelet volume; pITP, primary ITP; sITP, secondary ITP; TO, thiazole orange; TSC, trisodium citrate.

**FIGURE 3:**
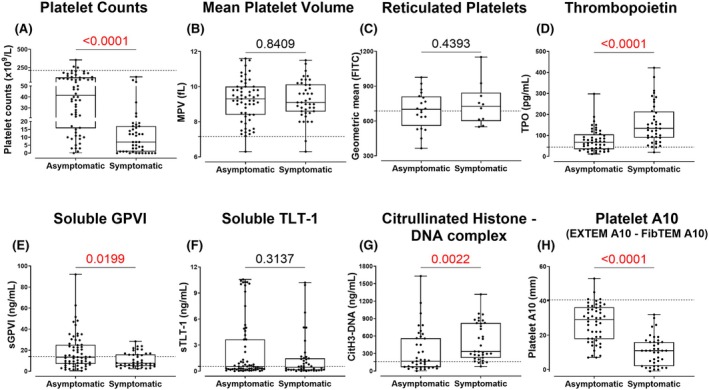
Platelet and plasma parameters in primary ITP patients with or without clinical symptoms of bleeding or bruising. (A) Platelet counts and (B) MPV were measured using an automated haematology analyser in TSC‐anti‐coagulated WB. (C) Reticulated platelets were quantified by TO staining in flow cytometry. Levels of (D) thrombopoietin (TPO), (E) soluble (s) GPVI, (F) sTLT‐1 and (G) citrullinated histone‐DNA (CitH3‐DNA) complex were measured by ELISA. (H) Platelet A10 was calculated by subtracting the FibTEM A10 measurement from the EXTEM A10 in ROTEM. The primary ITP patient cohort (*n* = 10–63) includes repeat measurements from the same subjects collected at different time points. An unpaired *t*‐test or Mann–Whitney test was performed depending on the distribution of the data. Significant *p*‐values (*p* < 0.05) are highlighted in red. The cut off for HD levels is indicated by dotted horizontal lines. ITP, immune thrombocytopenia; MPV, mean platelet volume; TO, thiazole orange; TSC, trisodium citrate.

**FIGURE 4:**
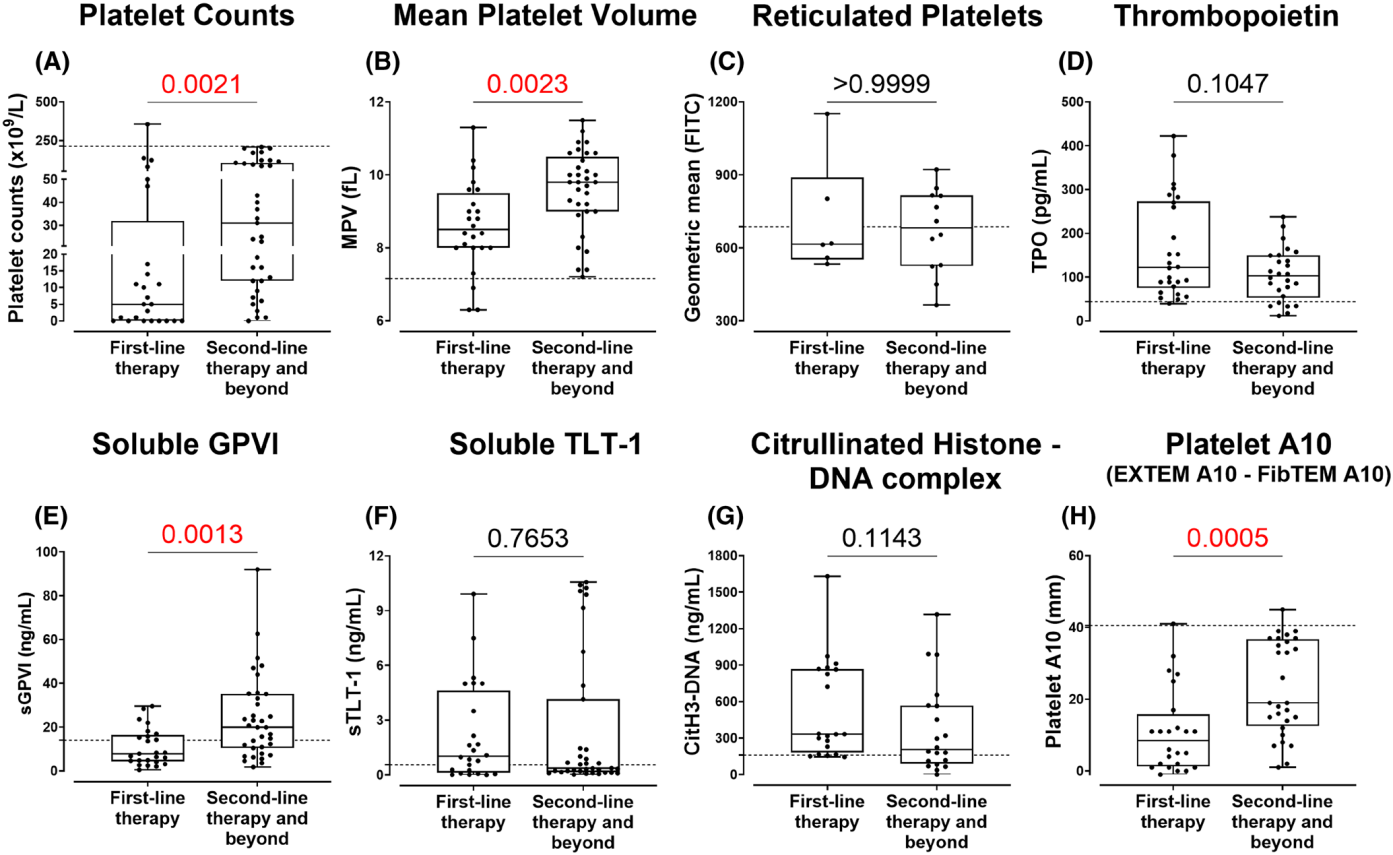
Platelet and plasma parameters in primary ITP patients stratified for therapy. Patients receiving first‐line therapy (steroids and/or IVIg) were compared to those receiving second‐line therapy and beyond (which include first line therapy plus treatments with TPO‐RA, rituximab, mycophenolate mofetil, dapsone, ciclosporin, danazol, vincristine, azathioprine, oseltamivir, ianalumumab and efgartigimod as well as splenectomy). (A) Platelet counts and (B) MPV were measured using an automated haematology analyser in TSC‐anti‐coagulated WB. (C) Reticulated platelets were quantified by flow cytometry. Levels of (D) TPO, (E) sGPVI, (F) sTLT‐1 and (G) CitH3‐DNA complexes were measured by ELISA. (H) Platelet A10 was calculated by subtracting the FibTEM A10 measurement from the EXTEM A10 in ROTEM. The primary ITP patient cohort (*n* = 6–35) includes repeat measurements from the same subjects collected at different time points. An unpaired *t*‐test or Mann–Whitney test was performed depending on the distribution of the data. Significant *p*‐values (*p* < 0.05) are highlighted in red. The cut‐off from HD levels is indicated by a dotted horizontal line on the respective graphs. ITP, immune thrombocytopenia; MPV, mean platelet volume; TPO‐RA, thrombopoietin receptor; TSC, trisodium citrate.

All patient groups (pITP, sITP and the control group) had significantly lower platelet counts (*p* < 0.0001 for all, Figure 2A) and increased mean platelet volume (MPV) (*p* < 0.0001, 0.0023 and *p* < 0.0001, respectively, Figure 2B) compared to healthy donors (HDs) and in patients receiving second‐line or beyond therapies (*p* = 0.0023; Figure 4B). Within the pITP cohort, platelet counts were significantly lower in symptomatic pITP (*p* < 0.0001, Figure 3A), newly diagnosed (*p* < 0.0489, Figure S3A) and in those receiving steroids and/or IVIg (*p* < 0.0021, Figure 4A). Although some patients with low platelet counts exhibited bleeding symptoms and others did not, statistical comparisons of MPV (Figure 3B and Figure S3B) and sTLT‐1 (Figure 3F and Figure S3F) between symptomatic and asymptomatic patients or with duration since diagnosis remained non‐significant even after adjusting for platelet count. This suggests that platelet count alone does not fully explain differences in bleeding manifestations, supporting the hypothesis that intrinsic platelet function contributes to bleeding risk in ITP^20^ newly released RNA‐enriched (TO^bright^) platelets showed no difference between HDs, ITP group and control group (Figure 2C) or across bleeding symptoms (Figure 3C), stage (Figure S3C) or first‐line treatments (Figure 4C). However, pITP patients showed a mild but significant correlation (*r* = 0.435, *p* = 0.030) between TO^bright^ events and surface GPIbα levels (Figure S4), aligning with higher GPIbα on younger platelets.^21^

### Serum TPO levels were elevated in symptomatic thrombocytopenic patients

The pITP, sITP and control groups had high serum TPO concentrations compared to HDs (*p* < 0.0001 for all, Figure 2D). In the pITP group, TPO levels showed a negative correlation with platelet count (*r* = −0.435, *p* < 0.0001, Figure S5A) and were significantly elevated in patients with platelet counts below 20 × 10^9^/L (Figure S5B) as well as in those experiencing bleeding or bruising at the time of collection (*p* < 0.0001; Figure 3D). No significant difference in TPO levels was observed between pITP patients receiving TPO receptor agonists and those on other treatments (Figure 4D and Figure S5C) or with duration since diagnosis (Figure S3D).

### Plasma sGPVI and sTLT‐1 in pITP

Elevated sGPVI levels were observed in asymptomatic pITP patients (*p* = 0.0199, Figure 3E) and in patients receiving second‐line line therapy and beyond (*p* = 0.0013, Figure 4E) but not sTLT‐1 (Figure 4F). No correlation was observed between sGPVI or sTLT‐1 with platelet counts (Figure S6B,D), duration since diagnosis (Figure S3E,F) or their respective surface receptors (Figure S6C,E), suggesting that proteolysis of these receptors was not constitutive and likely to be linked with platelet activation status. Consistent with this, a mild but significant correlation was found between sGPVI and sTLT‐1 levels (*r* = 0.302, *p* = 0.002, Figure S6A) in pITP samples. No differences in the soluble markers were observed in the pITP group when compared with HDs or the control group (Figure 2E,F); however, sITP showed elevated sGPVI when compared with pITP (*p* = 0.0059) and HDs (*p* = 0.0117) (Figure 2E).

### Surface GPVI and TLT‐1 levels were elevated in ITP patients with bleeding

Most of the platelet surface proteins remained unaltered in pITP patients when compared within subgroups; however, GPVI, TLT‐1 and anti‐human antibody binding were significantly elevated in patients exhibiting clinical symptoms (*p* = 0.0012, *p* = 0.0248 and *p* = 0.0029 respectively, Table 1). When compared with HDs, pITP patients showed increased expression of platelet activation markers P‐selectin and TLT‐1 (*p* = 0.0084 and *p* = 0.0136, respectively, Table S3) and anti‐human antibody binding (*p* = 0.0005, Table S3) despite the heterogeneity of the cohort.

**TABLE 1:** Platelet surface protein expression in primary ITP patients based on clinical presentation, disease chronicity and treatment.

Primary ITP^a^	Surface proteins changes in geometric mean (*p*‐value)								
	GPVI, *n* = 7–41	GPIbα, *n* = 7–41	Integrin αIIb, *n* = 7–41	Integrin α2, *n* = 6–27	CD9, *n* = 6–29	ADAM10, *n* = 6–28	P‐selectin, *n* = 6–28	TLT‐1, *n* = 7–38	Anti‐human IgG, *n* = 7–36
Symptoms (symptomatic vs. asymptomatic)									
*p*‐value	0.0012	0.9522	0.2066	0.5102	0.7733	0.5019	0.3460	0.0248	0.0029
Change	Increased	No change	No change	No change	No change	No change	No change	Increased	Increased
Stage (<3 months vs. >3 months)									
*p*‐value	0.8982	0.7227	0.1216	0.2686	0.8456	0.3635	0.8658	0.2893	0.3744
Change	No change	No change	No change	No change	No change	No change	No change	No change	No change
On first‐line treatment^b^ (Yes vs. No)									
*p*‐value	0.0654	0.9925	0.0541	0.2483	0.7378	0.8236	0.1505	0.0717	0.3760
Change	No change	No change	No change	No change	No change	No change	No change	No change	No change

Abbreviations: ADAM, a disintegrin and metalloproteinase; CD, cluster of differentiation; GP, glycoprotein; IgG, immunoglobulin G; ITP, immune thrombocytopenia; TLT‐1, TREM‐like transcript‐1.

^a^ Repeat measurements from the same patient on different days, including during recovery. An unpaired *t*‐test or Mann–Whitney test was performed depending on the distribution of the data. *p*‐values highlighted in red are significant (*p* < 0.05).

^b^ Patients receiving steroids and/or IVIg at the time of blood collection were classified as undergoing first‐line therapy, while the remaining patients were receiving second‐line or subsequent therapies.

### ITP patients demonstrated normal function of pathways controlling integrin αIIbβ3 activation

Platelets from pITP patients responded effectively to low and high doses of cross‐linked collagen‐related peptide (CRP‐xL) and adenosine diphosphate (ADP) in the OG‐Fg activation assay (Figure S2). There were no differences observed between HDs and pITP patients or within ITP subgroups.

### CitH3‐DNA complex levels were elevated in symptomatic ITP patients

Increased CitH3‐DNA complex levels were noted in bleeding or bruising pITP patients (*p* < 0.0333, Figure 2G and *p* < 0.0022, Figure 3G). The effect of therapy on CitH3‐DNA complex levels was investigated, and although the difference was not statistically significant (*p* = 0.1143, Figure 4G), higher levels were observed in ITP patients undergoing first‐line treatment. No significant correlation was observed between CitH3‐DNA complex levels and duration of diagnosis (Figure S3G) or platelet count (Figure S7A,B), indicating that elevated CitH3‐DNA levels in symptomatic patients were independent of platelet mass.

### ROTEM parameters can stratify ITP patients at greater risk of bleeding

**FIGURE 5:**
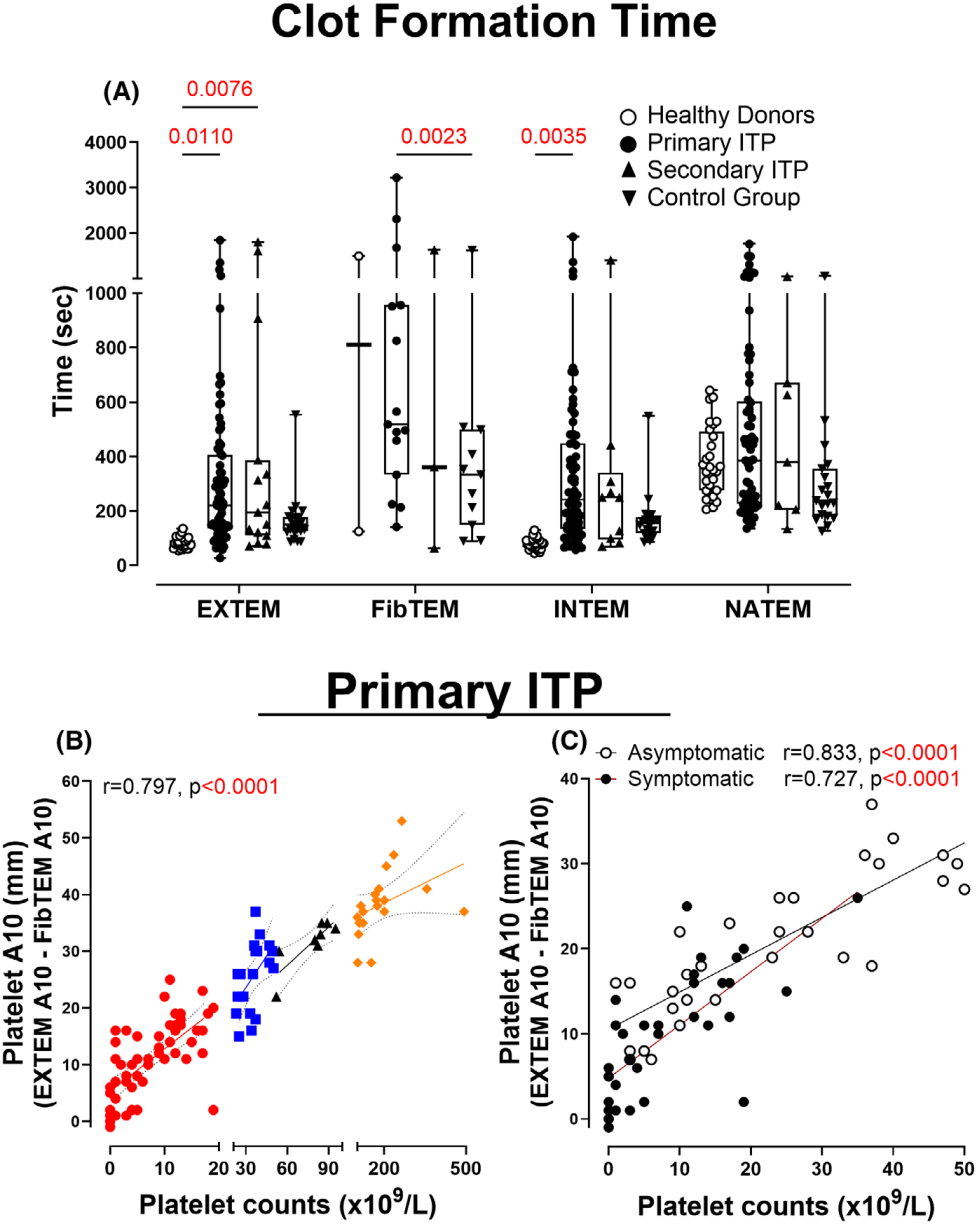
Platelet contribution to clot formation in primary ITP patients. (A) Clot formation time—time from initiation of clotting until a clot firmness of 20 mm—is detected. Platelet A10 was calculated by subtracting the FibTEM A10 measurement from the EXTEM A10. (B) Correlation between platelet A10 and platelet counts in ITP patients with platelet counts below 20 × 10^9^/L, 21–50 × 10^9^/L, 51–100 × 10^9^/L and over 100 × 10^9^/L was calculated. (C) Correlation between platelet A10 and platelet counts in primary ITP patients with (black circles) or without (open circles) symptoms of bleeding or bruising having a platelet count below 50 × 10^9^/L was calculated. Only significant *p*‐value (*p* < 0.05) highlighted in red are displayed. HDs (*n* = 2–28). The primary ITP patient cohort (*n* = 2–81) includes repeat measurements from the same subjects collected at different time points. Control group (*n* = 11–20) includes patients with platelet count below 100 × 10^9^/L due to causes other than ITP. Two‐way ANOVA with Bonferroni's multiple comparisons test was performed and the Pearson correlation coefficient (*r*) was calculated. HDs, healthy donors; ITP, immune thrombocytopenia.

Clot formation time (CFT) was significantly prolonged in pITP patients compared to HDs in EXTEM (*p* = 0.0099) and INTEM (*p* = 0.0032, Figure 5A). pITP samples also showed reduced clot amplitude at 10 min (A10) and maximum clot firmness (MCF) in EXTEM, INTEM and NATEM (Figure S8).

The platelet A10 was markedly reduced in all thrombocytopenic groups (all *p* < 0.0001, Figure 2H) and correlated strongly with platelet count (*r* = 0.797, *p* < 0.0001, Figure 5B) and was significantly lower in symptomatic patients (*p* < 0.0001, Figure 3H), those newly diagnosed (*p* = 0.0135, Figure S3H), or those receiving first‐line therapy (*p* = 0.0005, Figure 4H).

To determine whether the platelet contribution to the clot was compromised in pITP, patients with bleeding and bruising symptoms were compared to those with equivalent counts and no symptoms. Correlation curves were constructed of the platelet A10 versus platelet count in patients with platelet counts below 50 × 10^9^/L. Moderate correlation with clinical symptoms was observed (*r* = 0.727, *p* < 0.0001, Figure 5C). Using OLS linear regression, the symptomatic patients were found to have a significantly lower platelet A10 level relative to the platelet count than the non‐symptomatic patients (*p* = 0.0027).

### Multivariable analysis

Given the multifactorial nature of bleeding in ITP, a multivariable approach was used to identify key predictors and account for confounding among clinical and laboratory variables. The goal was to determine whether a combination of biomarkers could better predict bleeding risk than platelet count alone. Bleeding and bruising symptoms in ITP were modelled using logistic regression, incorporating significant variables (*p* < 0.05) in univariate analysis. To address multicollinearity and missing data, PPCA derived a composite score (principal component 1 [PC1]), explaining 42.6% variance (Table S4) and loading most strongly on platelet A10, TPO, GPVI, TLT‐1 and CitH3‐DNA (Table S5). Two logistic regression models were tested to predict the presence of symptoms in pITP (Table 2). In model 1, platelet count was used as the sole predictor of symptoms (*p* < 0.01, *β* = −0.98) but demonstrated limited sensitivity (53.5%) and accuracy (70.5%). Incorporating PC1 in model 2 improved model stability, accuracy (78.1% vs. 70.5%), sensitivity (69.8% vs. 53.5%) and AUC (0.87 vs. 0.83). PC1 emerged as the sole significant predictor (*p* = 0.002), while platelet count lost significance (*p* = 0.165), indicating a better overall fit (Figure S9).

**TABLE 2:** Multivariable logistic regression model and performance metrics.

Predictor	Model 1 (platelet count only)	Model 2 (PC1 included)
Intercept (SE, *p*‐value)	2.433 (0.611, *p* < 0.001)	0.701 (0.783, *p* < 0.001)
Platelet count (SE, *p*‐value)	−0.983 (0.201, *p* < 0.001)	−0.367 (0.264, *p* = 0.165)
PC1 (SE, *p*‐value)	—	1.194 (0.393, *p* = 0.002)
Sensitivity	0.535	0.698
Specificity	0.823	0.839
Positive predictive value	0.677	0.750
Negative predictive value	0.718	0.800
Accuracy % (95% CI)	70.48 (60.8–79.0)	78.1% (69–85.6)
Kappa	0.370	0.542
Area under curve (AUC)	0.83	0.87

Abbreviations: CI, confidence interval; PC1, principal component 1; SE, standard error.

## DISCUSSION

Patients with thrombocytopenia may experience bleeding events out of step with the platelet count for reasons which remain unclear, while other patients with severe thrombocytopenia may only experience minimal bleeding.^22^, ^23^ Current diagnostic and treatment approaches focus on thrombocytopenia rather than investigating and addressing platelet dysfunction, primarily due to a lack of reliable diagnostic tools to evaluate the pathology underlying thrombocytopenia. ITP is one such disease where, despite recent advances, platelet counts remain the principal determinant of clinical management with no definitive risk factors and no diagnostic or prognostic criteria.^23^, ^24^, ^25^ Here, we added a battery of research‐based approaches to explore changes in platelet activation, function and other indices and found increased surface GPVI and TLT‐1, increased serum TPO and plasma sGPVI, and evidence of increased NETosis in bleeding patients with pITP. Even allowing for platelet counts, there were also differences in the platelet contribution to clot formation in bleeding patients, implying a platelet dysfunction in pITP.

Subtle losses of receptor surface density can impact platelet activation and could underpin a mild bleeding propensity. Further, GPVI, TLT‐1 and GPIbα can be metalloproteolysed^26^ and were reduced in a heterogeneous ITP cohort.^27^ These proteins were selected, as we recorded an increased expression of GPVI and TLT‐1 in symptomatic patients that possibly suggest that their platelets were pro‐adhesive and pro‐aggregatory and contributed more to haemostasis compared to their asymptomatic counterparts. Although increased resting P‐selectin expression in ITP platelets is reported,^20^, ^23^ we did not observe a significant difference between patients with and without symptoms. This discrepancy may reflect differences in bleeding assessment methods, or technical aspects of P‐selectin measurement. Cohort differences, including a higher proportion of chronic cases and patients on corticosteroids, may blunt resting P‐selectin expression. Moreover, preferential clearance of activated platelets or shedding of P‐selectin into plasma could limit the sensitivity of surface measurements to distinguish clinical subgroups.

Although newly synthesised platelets were not elevated in the pITP cohort, a statistically significant positive relationship between GPIbα and the TO^bright^ population was found, consistent with links between GPIbα and circulating platelet age.^21^ This association likely reflects the preservation of GPIbα on younger, newly released platelets, which are less affected by receptor shedding or surface modulation that occurs as platelets age, activate, or with immune‐mediated damage.

Exposure of new surface receptors may compensate for platelet loss and reduced haemostatic capacity in ITP. However, OG‐Fg binding to activated platelets was stable across the subgroups. We used low and high doses of CRP‐xL to assess subtle changes in GPVI‐induced signalling, and PAR‐1 agonist and ADP were also used. pITP patients showed normal fibrinogen binding in response to all stimuli, indicating intact pathways regulating integrin αIIbβ3 activation.

This finding contrasts with previous reports describing impaired integrin activation in ITP platelets and an association with bleeding risk,^20^, ^28^ highlighting the heterogeneity of platelet dysfunction in this disorder. These discrepancies may reflect differences in assay methodologies, patient selection, cohort characteristics and sample size. Variations in agonist concentrations, flow cytometry gating strategies or sample preparation protocols can influence the assay sensitivity, while disease stage, severity and treatment regimens impact platelet function profiles. Thrombocytopenia mechanisms, whether driven by platelet destruction, impaired production or immune‐mediated functional alterations, may also result in distinct platelet phenotypes across studies.

Preserved integrin αIIbβ3 activation in our cohort suggests that compensatory mechanisms, including enhanced signalling through alternative pathways, may maintain platelet function despite low counts. This may explain why some patients with low platelet counts do not experience severe bleeding. These findings underscore that integrin dysfunction is not uniform in ITP and reinforce the need for standardised, mechanistically informed assays across diverse cohorts to clarify the relationship between platelet function and bleeding risk.

Changes in platelet receptor levels through proteolysis are associated with vascular injury^29^ and autoimmune disorders^30^, ^31^, ^32^ contributing to thrombocytopenia, bleeding and the generation of biospecific markers. Here plasma sGPVI and sTLT‐1 were measured. Elevated sGPVI in asymptomatic pITP patients compared to those with symptoms remains unexplained, but significant changes in patients on second‐line and subsequent therapies may aid treatment stratification. No variation in sTLT‐1 was observed by symptoms, disease duration, or treatment.

TPO levels in pITP were increased compared to HDs, aligning with other reported ranges.^33^ There was no variation in TPO levels based on the chronicity of the disease. However, elevated TPO levels were observed in patients who were symptomatic, had lower platelet counts and were receiving first‐line therapies for ITP. Furthermore, TPO levels in patients receiving TPO‐RA were unchanged. These drugs share no sequence homology with native TPO^34^ and are not detected in the TPO ELISA.

Recent studies have underscored the fundamental role of NETs in systemic autoimmune disorders.^35^ We found elevated NET complexes in samples from symptomatic pITP patients. This may indicate an accelerated autoimmune response in these patients, amplifying NET formation to promote further autoantigen generation, possibly fuelling ITP relapses and observed bleeding in our cohort. However, whether the autoimmune response triggers NET formation, or NETs formed via activated platelets initiate and perpetuate the immune response remains to be addressed.^36^ NETosis has been studied in prothrombotic states in ITP,^37^, ^38^ but its role in bleeding remains largely unexplored.

ROTEM offers insight into platelet and coagulation abnormalities with minimal sample processing.^39^, ^40^ As anticipated, ROTEM parameters that are sensitive to platelet numbers (CFT, MCF, A10 and α‐angle)^41^ were impaired in thrombocytopenic pITP samples. Correlations of bleeding score with clot firmness parameters in thromboelastography have been reported in adult and paediatric ITP.^42^, ^43^ Using a ROTEM parameters versus platelet count standard curve, we assessed differences in platelet function between samples that had equivalent low platelet numbers. Differential platelet A10 values in pITP samples with similar platelet counts were linked to bleeding incidence. These findings identify both a loss of ITP platelet haemostatic capacity and the utility of viscoelastometry to detect platelet dysfunction associated with bleeding in severe thrombocytopenic patients. However, analysis of ROTEM parameters in relation to other functional readouts revealed no significant correlations, suggesting that not all aspects of platelet dysfunction were captured by ROTEM alone. Platelet autoantibodies were also evaluated in relation to soluble receptor levels and platelet A10, but no significant correlations were observed.

We used PPCA to identify underlying data patterns and develop predictive models. PC1 incorporation improved model accuracy and increased sensitivity by reducing missed symptomatic cases. PC1 (comprising largely of platelet and vascular activation markers) functions as a composite score that captures complex platelet‐related mechanisms contributing to bleeding risk. Next steps will assess whether the model remains stable and generalisable across different patient populations. Further investigation into biological interpretations of PC1 may also provide insights into the underlying molecular mechanisms driving ITP bleeding risk. Most cases exhibited mild to moderate bleeding, which affects quality of life but does not strongly predict rare, life‐threatening events such as intracranial haemorrhage, as previously demonstrated.^44^, ^45^ Progression to major bleeding involves additional factors, including endothelial and coagulation integrity. Therefore, while bleeding assessments offer valuable clinical insight, they should be interpreted with caution when evaluating the risk of critical bleeding.

Conducting a study with patients referred from a hospital setting presents certain limitations and selection biases. Referrals were based on clinical indication rather than systematic enrolment, resulting in a cohort with diverse diagnoses, disease severities and treatment histories, reflecting real‐world clinical practice. Careful patient selection, informed consent, sample transport, concurrent healthy donor collection and periodic diagnosis reviews required a pragmatic approach. Most samples arrived at short notice and were promptly processed for multiple platelet experiments on the collection day. Despite the challenge, this enhanced the validity of findings. Although platelet function was analysed across treatments, no significant differences emerged; however, treatment heterogeneity remains a potential confounder for interpreting results and guiding future studies.

In conclusion, elevated surface GPVI and TLT‐1, serum TPO, plasma sGPVI and NETs, and intriguing differences in platelet contribution to clot formation in symptomatic patients support the presence of platelet dysfunction in pITP. Multivariable analysis showed that a composite measure of platelet parameters predicted bleeding risk more accurately than platelet count alone. These findings underscore the complexity of platelet dysfunction in ITP, where no single biomarker reliably predicts bleeding. By leveraging multivariable models and composite scores,^46^ the interplay between platelet function, activation and immune dysregulation using readily assayable vascular measures can be captured. These insights may improve risk stratification and guide personalised treatment, particularly as care shifts toward algorithm‐driven, targeted approaches. Further validation with independent datasets and prospective studies is needed to confirm clinical utility.

## AUTHOR CONTRIBUTIONS

SAA and SMH performed experiments, reviewed and interpreted the data, and drafted the initial manuscript; LAC conceived aspects of the study, performed experiments and reviewed and interpreted the data. SAB, VB, YLT and AK contributed to the acquisition of experimental data; RKA, EEG and PYIC conceived the study, acquired funding, reviewed the manuscript and interpreted the data. All authors reviewed the manuscript and approved its contents. PYIC received honoraria, speaking fees and travel assistance from Sobi, Novartis and Amgen; advisory boards for Janssen, Sanofi and Sobi; and research grants from Janssen and Novartis. EEG received speaking fees from Sobi.

## FUNDING INFORMATION

This work was supported by ACT Health (No. RIF2022001), International Society on Thrombosis and Haemostasis (No. ISTH RTWF 2019), National Blood Authority (No. NBA IgSO1) and National Health and Medical Research Council (Nos. APP2018835 and APP2000485).

## CONFLICT OF INTEREST STATEMENT

The authors have no conflicts of interest to disclose.

## Supporting information


Data S1.

## References

[1] Terrell DR , Beebe LA , Vesely SK , Neas BR , Segal JB , George JN . The incidence of immune thrombocytopenic purpura in children and adults: a critical review of published reports. Am J Hematol. 2010;85:174–180. 20131303 10.1002/ajh.21616

[2] Miltiadous O , Hou M , Bussel JB . Identifying and treating refractory ITP: difficulty in diagnosis and role of combination treatment. Blood. 2020;135:2325. 31756253 10.1182/blood.2019003599 PMC7484752

[3] Ghanima W , Gernsheimer T , Kuter DJ . How I treat primary ITP in adult who are unresponsive to or dependent on corticosteroid treatment. Blood. 2021;137:2736–2744. 33827138 10.1182/blood.2021010968

[4] Semple JW , Rebetz J , Maouia A , Kapur R . An update on the pathophysiology of immune thrombocytopenia. Curr Opin Hematol. 2020;27:423–429. 32868673 10.1097/MOH.0000000000000612

[5] Marini I , Bakchoul T . Pathophysiology of autoimmune thrombocytopenia: current insight with a focus on thrombopoiesis. Hamostaseologie. 2019;39:227–237. 30802916 10.1055/s-0039-1678732

[6] Scherlinger M , Richez C , Tsokos GC , Boilard E , Blanco P . The role of platelets in immune‐mediated inflammatory diseases. Nat Rev Immunol. 2023;23:495–510. 36707719 10.1038/s41577-023-00834-4 PMC9882748

[7] Vollenberg R , Jouni R , Norris PAA , Burg‐Roderfeld M , Cooper N , Rummel MJ , et al. Glycoprotein V is a relevant immune target in patients with immune thrombocytopenia. Haematologica. 2019;104:1237–1243. 30923095 10.3324/haematol.2018.211086 PMC6545841

[8] Quach ME , Dragovich MA , Chen W , Syed AK , Cao W , Liang X , et al. Fc‐independent immune thrombocytopenia via mechanomolecular signaling in platelets. Blood. 2018;131:787–796. 29203584 10.1182/blood-2017-05-784975 PMC5814932

[9] Choi PY‐I , Hicks S , Gardiner EE , Crispin P , Slade J , D'Rozario J , et al. Platelet dysfunction detected using rotational thromboelastometry (ROTEM) in severely thrombocytopenic patients with a bleeding phenotype. Blood. 2019;134:2357.

[10] Gardiner EE , Karunakaran D , Arthur JF , Mu F‐T , Powell MS , Baker RI , et al. Dual ITAM‐mediated proteolytic pathways for irreversible inactivation of platelet receptors: de‐ITAM‐izing FcgammaRIIa. Blood. 2008;111:165–174. 17848620 10.1182/blood-2007-04-086983

[11] Gardiner EE , Al‐Tamimi M , Mu FT , Karunakaran D , Thom JY , Moroi M , et al. Compromised ITAM‐based platelet receptor function in a patient with immune thrombocytopenic purpura. J Thromb Haemost. 2008;6:1175–1182. 18485087 10.1111/j.1538-7836.2008.03016.x

[12] Gardiner EE , Thom JY , Al‐Tamimi M , Hughes A , Berndt MC , Andrews RK , et al. Restored platelet function after romiplostim treatment in a patient with immune thrombocytopenic purpura. Br J Haematol. 2010;149:625–628. 20148887 10.1111/j.1365-2141.2010.08092.x

[13] Bayron‐Marrero Z , Branfield S , Menendez‐Perez J , Nieves‐López B , Ospina L , Cantres‐Rosario Y , et al. The characterization and evaluation of the soluble triggering receptor expressed on myeloid cells‐like transcript‐1 in stable coronary artery disease. Int J Mol Sci. 2023;24:13632. 37686440 10.3390/ijms241713632 PMC10487797

[14] Hidalgo A , Libby P , Soehnlein O , Aramburu IV , Papayannopoulos V , Silvestre‐Roig C . Neutrophil extracellular traps: from physiology to pathology. Cardiovasc Res. 2022;118:2737–2753. 34648022 10.1093/cvr/cvab329 PMC9586562

[15] Broome CM , Roth A , Kuter DJ , Scully M , Smith R , Wang J , et al. Safety and efficacy of classical complement pathway inhibition with sutimlimab in chronic immune thrombocytopenia. Blood Adv. 2023;7:987–996. 35973190 10.1182/bloodadvances.2021006864 PMC10027504

[16] Kuter DJ , Efraim M , Mayer J , Trněný M , McDonald V , Bird R , et al. Rilzabrutinib, an oral BTK inhibitor, in immune thrombocytopenia. N Engl J Med. 2022;386:1421–1431. 35417637 10.1056/NEJMoa2110297

[17] Tsykunova G , Holme PA , Tran HTT , Tvedt THA , Munthe LA , Michel M , et al. Daratumumab as a treatment for adult immune thrombocytopenia: a phase II study with safety run‐in (the DART study). Blood. 2021;138:2088.

[18] Liu X , Zhou H , Hu Y , Yin J , Li J , Chen W , et al. Sovleplenib (HMPL‐523), a novel Syk inhibitor, for patients with primary immune thrombocytopenia in China: a randomised, double‐blind, placebo‐controlled, phase 1b/2 study. Lancet Haematol. 2023;10:e406–e418. 37028433 10.1016/S2352-3026(23)00034-0

[19] Rodeghiero F , Michel M , Gernsheimer T , Ruggeri M , Blanchette V , Bussel JB , et al. Standardization of bleeding assessment in immune thrombocytopenia: report from the international working group. Blood. 2013;121:2596–2606. 23361904 10.1182/blood-2012-07-442392

[20] Frelinger AL , Grace RF , Gerrits AJ , Berny‐Lang MA , Brown T , Carmichael SL , et al. Platelet function tests, independent of platelet count, are associated with bleeding severity in ITP. Blood. 2015;126:873–879. 26138687 10.1182/blood-2015-02-628461 PMC4536541

[21] Andrews RK , Gardiner EE . Metalloproteolytic receptor shedding…Platelets “acting their age”. Platelets. 2016;27:512–518. 27459696 10.1080/09537104.2016.1212001

[22] Hicks SM , Coupland LA , Jahangiri A , Choi PY , Gardiner EE . Novel scientific approaches and future research directions in understanding ITP. Platelets. 2020;31:315–321. 32054377 10.1080/09537104.2020.1727871

[23] Mehic D , Machacek J , Schramm T , Buresch L , Kaider A , Eichelberger B , et al. Platelet function and soluble P‐selectin in patients with primary immune thrombocytopenia. Thromb Res. 2023;223:102–110. 36738663 10.1016/j.thromres.2023.01.012

[24] Choi PY , Merriman E , Bennett A , Enjeti AK , Tan CW , Goncalves I , et al. Consensus guidelines for the management of adult immune thrombocytopenia in Australia and New Zealand. Med J Aust. 2022;216:43–52. 34628650 10.5694/mja2.51284 PMC9293212

[25] Neunert C , Terrell DR , Arnold DM , Buchanan G , Cines DB , Cooper N , et al. American Society of Hematology 2019 guidelines for immune thrombocytopenia. Blood Adv. 2019;3:3829–3866. 31794604 10.1182/bloodadvances.2019000966 PMC6963252

[26] Gardiner EE , Karunakaran D , Shen Y , Arthur JF , Andrews RK , Berndt MC . Controlled shedding of platelet glycoprotein (GP)VI and GPIb‐IX‐V by ADAM family metalloproteinases. J Thromb Haemost. 2007;5:1530–1537. 17445093 10.1111/j.1538-7836.2007.02590.x

[27] Qiao J , Schoenwaelder SM , Mason KD , Tran H , Davis AK , Kaplan ZS , et al. Low adhesion receptor levels on circulating platelets in patients with lymphoproliferative diseases prior to receiving navitoclax (ABT‐263). Blood. 2013;121:1479–1481. 23429990 10.1182/blood-2012-12-467415

[28] Frelinger AL 3rd , Grace RF , Gerrits AJ , Carmichael SL , Forde EE , Michelson AD . Platelet function in ITP, independent of platelet count, is consistent over time and is associated with both current and subsequent bleeding severity. Thromb Haemost. 2018;118:143–151. 29304534 10.1160/TH17-06-0387

[29] Montague SJ , Delierneux C , Lecut C , Layios N , Dinsdale RJ , Lee CSM , et al. Soluble GPVI is elevated in injured patients: shedding is mediated by fibrin activation of GPVI. Blood Adv. 2018;2:240–251. 29437639 10.1182/bloodadvances.2017011171 PMC5812322

[30] Nurden P , Tandon N , Takizawa H , Couzi L , Morel D , Fiore M , et al. An acquired inhibitor to the GPVI platelet collagen receptor in a patient with lupus nephritis. J Thromb Haemost. 2009;7:1541–1549. 19583823 10.1111/j.1538-7836.2009.03537.x

[31] Pishko AM , Andrews RK , Gardiner EE , Lefler DS , Cuker A . Soluble glycoprotein VI is a predictor of major bleeding in patients with suspected heparin‐induced thrombocytopenia. Blood Adv. 2020;4:4327–4332. 32915974 10.1182/bloodadvances.2020002861 PMC7509866

[32] Rabbolini DJ , Gardiner EE , Morel‐Kopp MC , Dunkley S , Jahangiri A , Lee CSM , et al. Anti‐glycoprotein VI mediated immune thrombocytopenia: an under‐recognized and significant entity? Res Pract Thromb Haemost. 2017;1:291–295. 30046699 10.1002/rth2.12033 PMC6058269

[33] Makar RS , Zhukov OS , Sahud MA , Kuter DJ . Thrombopoietin levels in patients with disorders of platelet production: diagnostic potential and utility in predicting response to TPO receptor agonists. Am J Hematol. 2013;88:1041–1044. 23913253 10.1002/ajh.23562

[34] Provan D , Semple JW . Recent advances in the mechanisms and treatment of immune thrombocytopenia. EBioMedicine. 2022;76:103820. 35074629 10.1016/j.ebiom.2022.103820 PMC8792416

[35] Wigerblad G , Kaplan MJ . Neutrophil extracellular traps in systemic autoimmune and autoinflammatory diseases. Nat Rev Immunol. 2023;23:274–288. 36257987 10.1038/s41577-022-00787-0 PMC9579530

[36] Nelson VS , Jolink AC , Amini SN , Zwaginga JJ , Netelenbos T , Semple JW , et al. Platelets in ITP: victims in charge of their own fate? Cells. 2021;10:3235–3250. 34831457 10.3390/cells10113235 PMC8621961

[37] Garabet L , Henriksson CE , Lozano ML , Ghanima W , Bussel J , Brodin E , et al. Markers of endothelial cell activation and neutrophil extracellular traps are elevated in immune thrombocytopenia but are not enhanced by thrombopoietin receptor agonists. Thromb Res. 2020;185:119–124. 31805421 10.1016/j.thromres.2019.11.031

[38] Lozano ML , Garabet L , Fernandez‐Perez MP , de Los Reyes‐García AM , Diaz‐Lozano P , Garcia‐Barbera N , et al. Platelet activation and neutrophil extracellular trap (NET) formation in immune thrombocytopenia: is there an association? Platelets. 2020;31:906–912. 31762368 10.1080/09537104.2019.1696456

[39] Alessi MC , Sie P , Payrastre B . Strengths and weaknesses of light transmission aggregometry in diagnosing hereditary platelet function disorders. J Clin Med. 2020;9:9. 10.3390/jcm9030763 PMC7141357 32178287

[40] Kelly JM , Rizoli S , Veigas P , Hollands S , Min A . Using rotational thromboelastometry clot firmness at 5 minutes (ROTEM((R)) EXTEM A5) to predict massive transfusion and in‐hospital mortality in trauma: a retrospective analysis of 1146 patients. Anaesthesia. 2018;73:1103–1109. 29658985 10.1111/anae.14297 PMC6120456

[41] Drotarova M , Zolkova J , Belakova KM , Brunclikova M , Skornova I , Stasko J , et al. Basic principles of rotational thromboelastometry (ROTEM((R))) and the role of ROTEM‐guided fibrinogen replacement therapy in the management of coagulopathies. Diagnostics (Basel). 2023;13:3219–3233. 37892040 10.3390/diagnostics13203219 PMC10606358

[42] Greene LA , Chen S , Seery C , Imahiyerobo AM , Bussel JB . Beyond the platelet count: immature platelet fraction and thromboelastometry correlate with bleeding in patients with immune thrombocytopenia. Br J Haematol. 2014;166:592–600. 24797389 10.1111/bjh.12929

[43] Gunduz E , Akay OM , Bal C , Gulbas Z . Can thrombelastography be a new tool to assess bleeding risk in patients with idiopathic thrombocytopenic purpura? Platelets. 2011;22:516–520. 21557684 10.3109/09537104.2011.571317

[44] Lambert C , Maitland H , Ghanima W . Risk‐based and individualised management of bleeding and thrombotic events in adults with primary immune thrombocytopenia (ITP). Eur J Haematol. 2024;112:504–515. 38088207 10.1111/ejh.14154

[45] Hato T , Shimada N , Kurata Y , Kuwana M , Fujimura K , Kashiwagi H , et al. Risk factors for skin, mucosal, and organ bleeding in adults with primary ITP: a nationwide study in Japan. Blood Adv. 2020;4:1648–1655. 32320469 10.1182/bloodadvances.2020001446 PMC7189281

[46] Chuah A , Hewitt TC , Ali SA , May M , Xu T , Christiadi D , et al. EDAmame: interactive exploratory data analyses with explainable models. Bioinformatics. 2025;41:btaf340. 40579229 10.1093/bioinformatics/btaf340 PMC12205176

