# Supplementary material for: Platelet dysfunction in immune thrombocytopenia: Finding clinical subsets with platelet phenotypes
Source: Br J Haematol. 2025 Sep 29;208(1):274–84. doi: 10.1111/bjh.70179 (PMC12819099; doi:10.1111/bjh.70179)
Supplement: Supplementary file 1 — Data S1. [file BJH-208-274-s001.docx]

**Platelet dysfunction in immune thrombocytopenia: finding clinical subsets with platelet phenotypes**

Sidra A. Ali,^1*^ Sarah M. Hicks,^1*^ Lucy A. Coupland,^2,3^ Simone A. Brysland,^1^ Vijay Bhoopalan,^1^ Yee Lin Thong,^1^ Amandeep Kaur,^1^ Robert K. Andrews,^3^ Elizabeth E. Gardiner^1,3†^ and Philip Y-I. Choi^1,3,4†^

**Supplementary Materials and Methods**

NPRC patient cohort

Diagnoses made by treating physicians were subject to revision as new clinical information became available over time. Three patients were reclassified as having Evans syndrome and categorised as secondary ITP. Thirteen patients who were initially suspected of having ITP were later found to have alternative causes of thrombocytopenia, including underlying liver disease, drug-induced thrombocytopenia, inherited platelet disorders, or cancer-related causes, and were reassigned to the control group. This approach ensured that final group allocations reflected the most accurate clinical diagnoses and maintained the robustness of group comparisons. Clinical histories, including bleeding assessments, comorbidities, medication use (such as NSAIDs or other agents known to affect platelet function) and thrombotic events were performed by the treating clinical team at sample collection. Documented arterial or venous thrombosis was present in 6/70 (8.5%) patients with pITP and 2/11 (18.1%) patients with sITP. While the overall frequency of thrombosis aligned with previously reported rates in ITP populations^1^, correlation analysis between thrombotic events and platelet parameters could not be reliably performed due to the limited number of events. Similarly, the utility of bleeding scores for biomarker correlation was limited by the narrow range of bleeding severity in the cohort, with most patients exhibiting only minor bleeding (score of 1). Accordingly, bleeding was analysed as a binary outcome (presence *versus* absence), and logistic regression models were used to evaluate associations between biomarker panels and bleeding symptoms. Samples were collected at the time of referral. To minimise sample processing and platelet loss, most experiments were carried out using anticoagulated WB, following a protocol designed to maximise platelet evaluation, alongside HD samples collected on same day.

Blood drawing

This study received Human Research Ethics Committee approval from ANU (2022/372 and 2017/924) and ACT Health (ETHLR.18.058). All patients and healthy donors (HDs) provided informed consent prior to participation, and the study was conducted as per the Declaration of Helsinki under the NPRC protocol at The Canberra Hospital. HD blood was drawn using a 19G winged infusion set into syringes containing 3.2% trisodium citrate (TSC), 5 mM ethylenediaminetetraacetic acid (EDTA), or without anticoagulants for serum isolation. Patient blood was drawn into vacutainers containing TSC or EDTA (pink and white top tubes). Whole blood (WB) cell counts were obtained using an automated haematological analyser (Cell-Dyn Emerald 22, Abbott Diagnostics, USA). Blood samples were processed as soon as possible after collection, typically within 1-4 h, to minimise artefactual platelet activation and preserve physiological function.

Reagents

Collagen-related peptide (CRP) monomer was purchased (Auspep, Melbourne, Australia) and chemically cross-linked as described.^2^ Thiazole orange (TO), trisodium citrate (TSC), adenosine diphosphate (ADP), ethylenediaminetetraacetic acid (EDTA), bovine serum albumin (BSA) and TWEEN®-20 were from Sigma-Aldrich (Merck, Darmstadt, Germany). Oregon green-488 fibrinogen (OG-Fg) was from Thermo Fisher (Waltham, MA, USA), PAR-1 agonist TRAP-6 was from R&D Systems (Minneapolis, MN, USA) and ROTEM reagents were from Werfen (Sydney, Australia). All other reagents were of laboratory grade.

Antibodies

Phycoerythrin (PE) conjugated anti-αIIb monoclonal antibody (mAb), clone MEM-06, PE conjugated anti-CD62P mAb, clone AK4, and fluorescein isothiocyanate (FITC) conjugated anti-human IgG polyclonal antibody were sourced from Abcam (Melbourne, Australia). Allophycocyanin (APC)-conjugated anti-ADAM10, clone 163003, PE-conjugated anti-CD9, clone 209306, and FITC-conjugated anti-Trem-like Transcript (TLT)-1 mAbs, clone 268420 were obtained from R&D Systems (Minneapolis, MN, USA). FITC-conjugated anti-GPIbα mAb, clone AK2, was from Invitrogen (Thermo Fisher Scientific, Waltham, MA, USA), APC-conjugated anti-α2 integrin subunit mAb, clone P1E6-C5, was from BioLegend (San Diego, CA, USA), and FITC-conjugated anti-human IgG polyclonal antibodies were from eBioscience (California, USA). Anti-GPVI mAbs, clones 1A12 and12C9 and FITC-conjugated clone 1G5, have been previously described.^3^

Reticulated Platelets

TSC-anticoagulated WB was diluted to a platelet count of 5 x 10^7^/mL in 137 mM NaCl containing 2.7 mM KCl, 10 mM Na2HPO4, 1.8 mM KH2PO4, 2 mM EDTA, pH 7.4 (phosphate buffered saline (PBS)-EDTA). Blood samples with a platelet count below 5 x 10^7^/mL were not diluted. Methanol-dissolved Thiazole orange (TO) was diluted to 0.5 ug/mL in PBS-EDTA then mixed with blood samples to a final concentration of 50 ng/mL. Samples were incubated at RT for 1.5 h in the dark then mixed with PE-anti-αIIb (1:250) and further incubated for 30 min. 5000 platelet events were acquired on a FACSCalibur. A gate collected the top 10% of CD41- and TO-positive events (TO^bright^) in a HD sample processed in parallel with each patient; the same gate was then applied to patient samples. The geometric mean of TO fluorescence was determined within this gated population. Recent studies have questioned the specificity of TO for identifying reticulated platelets due to potential confounding by activated platelets, large platelets, or microparticles^4,5^ We ensured reliable assessment of reticulated platelets by using stringent gating to minimise non-specific signals and a healthy donor with each patient sample.

Resting and activated platelet αIIbβ3 measurement using OG-Fg

80 µL of TSC-anticoagulated WB was treated with 0.5 or 5 μg/mL CRP-xL, 10 µM thrombin receptor-activating peptide (TRAP)-6, or 5 μM ADP in the presence of 15 µg/mL of OG-Fg at RT for 2 h, protected from light. The 2 h incubation was selected to synchronise the completion of multiple platelet assays run in parallel, allowing all tests to conclude at a similar time and enabling simultaneous sample acquisition. After incubation, reactions were stopped by the addition of 50 mM EDTA and diluted 1:10 with 10 mM Tris-HCl pH 7.4, containing 150 mM NaCl and 5 mM EDTA (TS-EDTA). EDTA was added only after the activation and binding steps to terminate the reaction and prevent further platelet activation or aggregation; calcium was present during the binding phase to allow physiological fibrinogen-αIIbβ3 interaction. Fibrinogen binding to assess integrin αIIbβ3 activation was measured by the extent of fluorescence activity of OG-Fg using flow cytometry. This method directly measures the functional ligand-binding capacity of αIIbβ3, providing a physiologically relevant assessment. Platelets were gated using forward and side scatter properties and levels of OG-Fg binding across 5000 platelet events were assessed using the FL-1 channel. Positive OG-Fg gate was defined as fluorescence activity greater than that of unstimulated healthy donor samples. The same gate was applied to all other samples.

Evaluation of platelet surface proteins

WB was used, which is well-suited for patients with low platelet counts and minimises sample manipulation. TSC-anticoagulated WB was diluted 1:5 with TS-EDTA and incubated with fluorescently conjugated antibodies against platelet-specific proteins at RT for 30 min, protected from light. Samples were diluted 2.5-fold with TS-EDTA, and antibody binding to 5000 platelet events (see Supplementary Figure S1 for gating strategy) was quantified on a FACSCalibur.

Soluble GPVI ELISA

The assay was optimised using monoclonal mouse anti-human GPVI capture and detection antibody. Briefly, a 96-well plate (MaxiSorp, NUNC Denmark) coated with 12C9^3^ mAb against human GPVI, overnight at 4°C, was washed with PBS containing 0.2% TWEEN®-20 (PBS-T) and blocked with 1% (w/v) BSA. Duplicate aliquots of double-spun platelet-free plasma (PPP) were added for 1 h at RT. The plate was again washed with PBS-T and incubated with HRP-conjugated anti-human GPVI mAb 1A12. Bound antibody was quantified using enhanced chemiluminescence in a plate reader (Tecan Infinite 200Pro, Switzerland) by adding SuperSignal ELISA substrate (Pierce, Rockford, IL, USA) for 1 min. Amounts of sGPVI were interpolated against a standard curve constructed by mixing increasing amounts of N-ethylmaleimide (NEM)-treated platelet-rich plasma (PRP) with GPVI-depleted plasma.

Measurement of serum and other plasma proteins

Serum thrombopoietin (TPO) levels were measured by ELISA (R&D Systems DTP00B). sTLT-1 levels were measured in platelet-free plasma by ELISA (R&D Systems DY2394). NETs were quantified by ELISA in plasma isolated from WB collected in a cell-free DNA collection tube containing EDTA (Roche 07785674001) by measuring levels of CitH3-DNA complexes following published methods.^6^

Rotational thromboelastometry (ROTEM) for the evaluation of clot formation

Viscoelastography analysis of WB clotting parameters was performed using a ROTEM delta device (Werfen, Sydney, Australia) according to manufacturer’s instructions. The NATEM (using CaCl_2_ only), EXTEM (using CaCl_2_ and tissue factor), INTEM (using CaCl_2_, phospholipid, and ellagic acid), and FibTEM (using tissue factor and cytochalasin D) tests were performed with TSC-anticoagulated WB within 4 h of phlebotomy.

Statistical analysis

Normally distributed data were analysed using unpaired t-tests or one-way analysis of variance (ANOVA) with Bonferroni’s multiple comparisons correction. Normality of residuals was assessed using D’Agostino and Pearson, Anderson-Darling, and Shapiro-Wilk tests. Non-normally distributed data were analysed using the Mann-Whitney U test or Kruskal-Wallis test with Dunn’s post hoc multiple comparisons test. Box and whisker plots illustrated minimum, median, and maximum values, along with the first and third interquartile ranges. Associations between platelet count and platelet A10 level in pITP patients with and without bleeding, was assessed using ordinary least squares (OLS) regression. The interaction between bleeding status and the association between platelet count and platelet A10 level was tested by incorporating an interaction term. Variables with p<0.05 in univariate analyses underwent logistic regression (symptoms as outcome) and continuous variables were log-transformed. Probabilistic principal component analysis (PPCA)^7^ addressed multicollinearity, imputed missing data, and derived composite scores. Model performance was assessed via ROC analysis (caret package).^8^ Analyses used R (v4.3.1)^9^ and GraphPad Prism (v10.2.1). Statistical significance was set at α=0.05, and Pearson correlation coefficient (r) was used to assess the strength of linear associations.

**References**

1. Goncalves I, Lewis C, Grainger B, et al. Thrombosis in patients with immune thrombocytopenia: incidence, risk, and clinical outcomes. Res Pract Thromb Haemost. 2024;8:102342.

2. Morton LF, Hargreaves PG, Farndale RW, Young RD, Barnes MJ. Integrin α2β1-independent activation of platelets by simple collagen-like peptides: collagen tertiary (triple-helical) and quaternary (polymeric) structures are sufficient alone for α2β1-independent platelet reactivity. Biochem J. 1995;306 ( Pt 2):337-344.

3. Al-Tamimi M, Mu FT, Arthur JF, et al. Anti-glycoprotein VI monoclonal antibodies directly aggregate platelets independently of FcγRIIa and induce GPVI ectodomain shedding. Platelets. 2009;20:75-82.

4. Reddy EC, Wang H, Bang KWA, Packham MA, Rand ML. Young steady-state rabbit platelets do not have an enhanced capacity to expose procoagulant phosphatidylserine. Platelets. 2018;29:27-33.

5. Angenieux C, Couvidou A, Brouard N, et al. Discriminating young platelets on human leukocyte antigen-I expression highlights their extremely high reactivity potential. Res Pract Thromb Haemost. 2023;7:100006.

6. Thalin C, Aguilera K, Hall NW, et al. Quantification of citrullinated histones: Development of an improved assay to reliably quantify nucleosomal H3Cit in human plasma. J Thromb Haemost. 2020;18:2732-2743.

7. Stacklies W, Redestig H, Scholz M, Walther D, Selbig J. pcaMethods--a bioconductor package providing PCA methods for incomplete data. Bioinformatics. 2007;23:1164-1167.

8. Kuhn M. Building Predictive Models in R Using the caret Package. Journal of Statistical Software. 2008;28:1-26.

9. R Core Team. R: A language and environment for statistical computing: R Foundation for Statistical Computing; <https://www.R-project.org/>; 2023.

**Supplementary Tables:**

**Table S1:** **Primary ITP classification based on** **clinical presentation and treatment.**

| **Primary ITP subgroup*** | **Newly Diagnosed**  **n=33** | **Persistent**  **n=8** | **Chronic**  **n=69** |
| --- | --- | --- | --- |
| **Bleeding/bruising^†^** |  |  |  |
| Yes (%) | 19 (58) | 4 (50) | 21 (30) |
| No (%) | 14 (42) | 4 (50) | 48 (70) |
| **On treatment^†^** |  |  |  |
| Yes (%) | 20 (61) | 4 (50) | 40 (58) |
| No (%) | 13 (39) | 4 (50) | 29 (42) |
| **On first line therapy**^‡^ |  |  |  |
| Yes (%) | 17 (52) | 3 (38) | 7 (10) |
| No (%) | 03 (9) | 1 (13) | 33 (48) |

*****110 encounters from primary ITP patients, involving 70 individual patients, with repeated sample collections (n=21) at different time points, including during remission.

**^†^**Treatment includes steroids, IVIg, TPO-RA, rituximab, mycophenolate mofetil, dapsone, cyclosporine, danazol, vincristine, azathioprine, oseltamivir, ianalumumab, efgartigimod and/or splenectomy at time of blood collection.

^‡^ Primary ITP patients receiving steroids and/or IVIg at the time of blood collection were classified as undergoing first-line therapy, while the remaining patients receiving other treatments were categorised as receiving second- or subsequent lines of therapy.

| Likely causes of secondary ITP | n=11 |
| --- | --- |
| Drug induced* | 2 |
| Systemic lupus erythematosus | 2 |
| Evans Syndrome | 2 |
| ChAdOx1 vaccine-induced | 1 |
| STAT3 gain of function | 1 |
| Sjögren's syndrome | 1 |
| Paraneoplastic | 1 |
| Inflammatory bowel disease | 1 |
| **Likely causes of thrombocytopenia in control group** | **n=22** |
| Cancer related^†^ | 6 |
| Liver disease^‡^ | 4 |
| RUNX1 associated | 2 |
| Drug induced^§^ | 1 |
| Inherited thrombocytopenia^#^ | 2 |
| Other^ | 7 |

**Table S2: Causes of thrombocytopenia in secondary ITP and control group.**

*Includes alemtuzumab and a checkpoint inhibitor.

^†^Includes acute myeloid leukemia, breast cancer, myelodysplastic syndrome, suspected gastrointestinal malignancy, thymoma and leiomyosarcoma.

^‡^Includes fatty liver disease, cirrhosis, hemi-hepatectomy and non-alcoholic steatohepatitis.

^§^Includes ruxolitinib and piperacillin / tazobactam.

**^#^** Includes grey platelet syndrome and MYH9 platelet disorder.

**^**Includes aplastic anemia, nutritional deficiency, bacterial (LPS)-induced and gestational thrombocytopenia.

**Table S3: Platelet surface molecule expression in healthy donors, ITP and control group.**

| **Surface proteins** | **Healthy Donors** | | **Primary ITP** | **Secondary ITP** | | **Control group** | **P-value^†^** |
| --- | --- | --- | --- | --- | --- | --- | --- |
| **Adheso-signalling receptors** | | | | | | | |
| **GPVI** | n=32 | | n=56 | n=9 | n=12 | |  |
| Median | 479.5  (391.8 - 551.8) | | 474.0  (402.3 - 581.3) | 528.0  (381.5 - 641.5) | | 319.0  (212.5-510.5) |  |
| P-value**^#^** | - | | >0.999 | >0.999 | | 0.321 | 0.0792 |
| **GPIbα** | n=32 | | n=55 | n=9 | n=12 | |  |
| Median | 1495  (1281 -1649) | | 1614  (1364 - 1894) | 1508  (1286 - 1757) | | 1444  (1320 - 1737) |  |
| P-value**^#^** | - | | 0.409 | >0.999 | | >0.999 | >0.9999 |
| **Stable surface markers** | | | | | | | |
| **αIIb integrin** | n=32 | | n=56 | n=9 | n=12 | |  |
| Median | 1792  (1552 - 1984) | | 1755  (1372 - 2018) | 1785  (1483 - 2232) | | 1917  (1727 - 2496) |  |
| P-value**^#^** | - | | >0.999 | >0.999 | | 0.758 | 0.2406 |
| **α2 integrin** | n=27 | | n=39 | n=4 | n=11 | |  |
| Median | 57.20  (39.30 - 75.80) | | 65.20  (33.0 - 84.80) | 79.10  (45.15 - 100.0) | | 52.00  (39.70 - 63.50) |  |
| P-value**^#^** | - | | >0.999 | >0.999 | | >0.999 | >0.9999 |
| **CD9** | n=30 | | n=43 | n=5 | n=12 | |  |
| Median | 20.65  (17.08 - 25.25) | | 20.90  (12.50 - 29.30) | 31.20  (7.72 - 46.60) | | 26.85  (23.13 - 33.13) |  |
| P-value**^#^** | - | | >0.999 | >0.999 | | 0.068 | 0.0688 |
| **ADAM10** | n=30 | | n=42 | n=5 | n=12 | |  |
| Median | 31.20  (19.58 - 39.58) | | 33.05  (21.00 - 44.90) | 39.20  (26.20 - 51.00) | | 38.60  (23.20 - 42.05) |  |
| P-value**^#^** | - | | >0.999 | >0.999 | | >0.999 | >0.9999 |
| **Platelet activation markers** | | | | | | | |
| **P-selectin** | n=28 | | n=40 | n=4 | | n=11 |  |
| Median | 5.520  (3.35 - 12.32) | | 13.40  (6.55 - 22.55) | 14.10  (6.17 - 17.78) | | 12.30  (4.90 - 32.40) |  |
| P-value**^#^** | - | | 0.0084 | >0.9999 | | 0.4424 | >0.9999 |
| **TLT-1** | n=30 | | n=53 | n=8 | n=11 | |  |
| Median | 78.60  (60.50 - 124.8) | | 159.0  (89.80 - 241.0) | 190.0  (79.80 - 206.5) | | 182.0  (97.30 - 338.0) |  |
| P-value**^#^** | - | | 0.0136 | 0.3434 | | 0.0250 | >0.9999 |
| **Anti-human antibody binding** | | n=21 | n=47 | n=8 | n=9 | |  |
| Median | 8.89  (6.55- 10.90) | | 16.50  (9.95 - 25.80) | 14.80  (10.85 - 31.70) | | 23.80  (18.05 - 30.80) |  |
| P-value**^#^** | - | | 0.0005 | 0.0713 | | 0.0003 | 0.6301 |

Data are expressed as median (25^th^-75^th^ percentile) and includes repeat measurements from the same patient on different days, including during recovery period. Ordinary one-way ANOVA Bonferroni's multiple comparisons test or a Kruskal-Wallis with Dunn’s multiple comparisons statistical tests were performed depending on distribution of the data. Significant p-values (p<0.05) are highlighted in red. †Between primary ITP and control group. #Between patient group and healthy donors. ITP=immune thrombocytopenia, control group=patients with platelet count below 100 x 10^9^/L due to causes other than ITP, GP=glycoprotein, CD=cluster of differentiation, ADAM=a disintegrin and metalloproteinase, TLT-1=TREM like transcript-1

**Table S4: Variance explained by principal components.**

|  | PC1 | PC2 | PC3 |
| --- | --- | --- | --- |
| R^2^ | 0.4257 | 0.2093 | 0.1406 |
| Cumulative R^2^ | 0.4257 | 0.6349 | 0.7755 |

R^2^=coefficient of determination, PC=principal component

**Table S5: PCA component loadings and composite scoring.**

|  | **PC1** | **PC2** | **PC3** |
| --- | --- | --- | --- |
| TPO | -0.541 | -0.334 | -0.183 |
| GPVI | -0.363 | 0.032 | -0.013 |
| TLT-1 | -0.306 | -0.011 | 0.019 |
| CitH3-DNA | -0.139 | -0.245 | 0.956 |
| sGPVI | 0.282 | -0.906 | -0.180 |
| Platelet A10 | 0.619 | 0.079 | 0.139 |


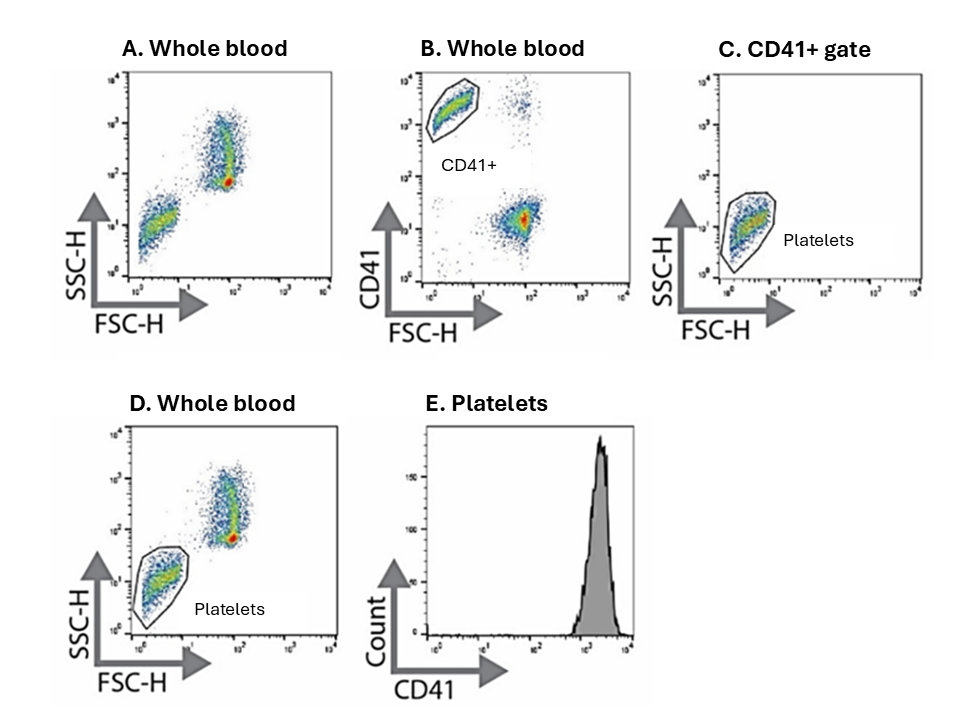
**Supplementary Figures:**

Figure S1: Gating strategy for platelet surface receptor enumeration.

Flow cytometric data were acquired on a BD FACSCalibur. **A.** WB forward and side scatter are on x and y axes, respectively. **B.** The platelet gate is drawn around the CD41^+^ events only. **C.** Another gate is created around this population using forward and side scatter to be used for platelet events recorded with non-CD41 antibodies conjugated to various fluorophores. **D.** The new gate using forward and side scatter is applied to the WB. **E.** To validate that all events inside the platelet gate are CD41^+^, the platelet gate is assessed in a CD41^+^ histogram. The gating strategy was deemed satisfactory if at least 2000 events within the platelet gate were CD41^+^. FSC-H=forward scatter height, SSC-H=side scatter height, CD=cluster of differentiation.


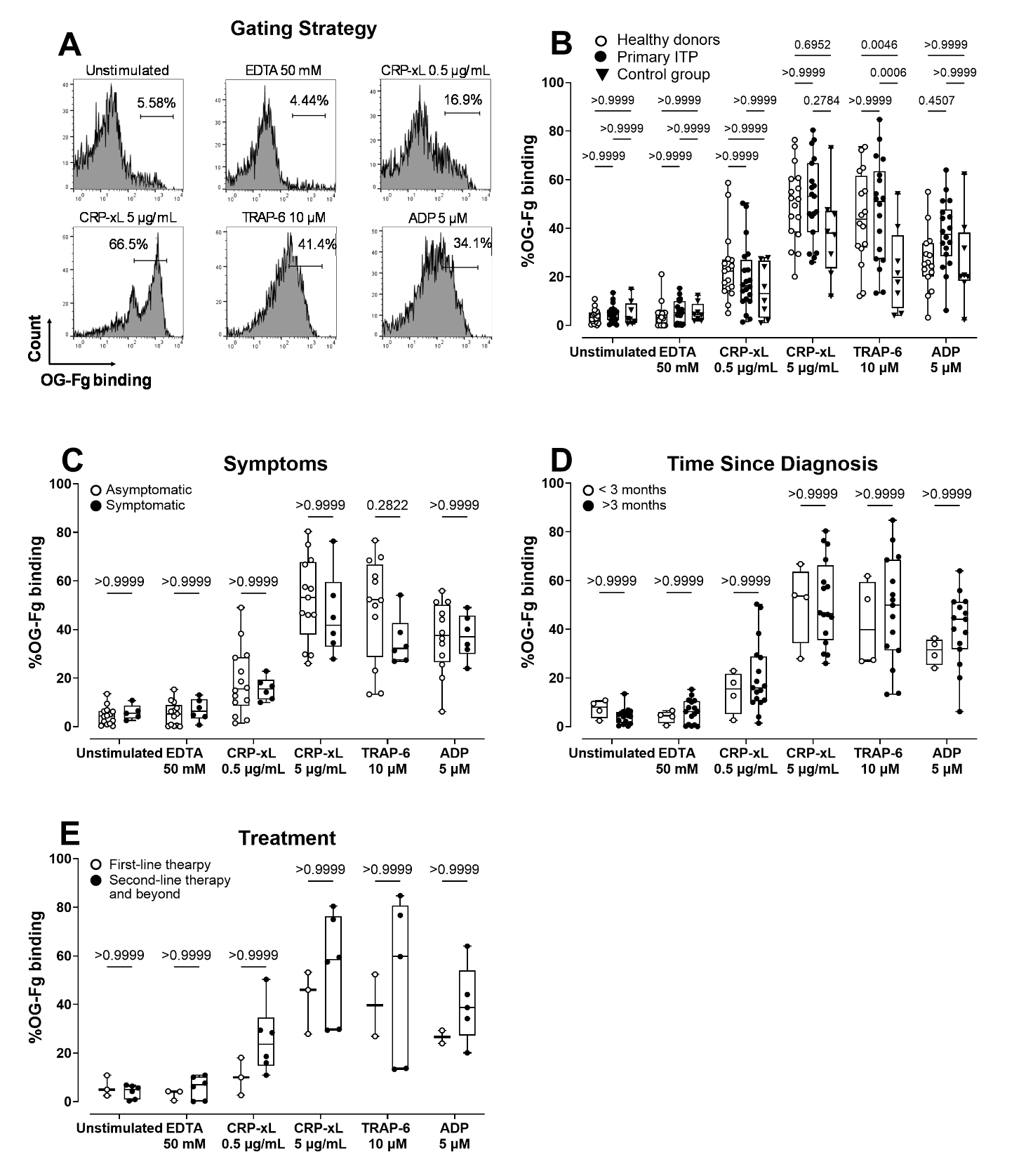
**Figure S2: OG-Fg binding in healthy donors, primary ITP and control group.**

OG-Fg binding was measured by flowcytometry **A.** TSC-anticoagulated WB was activated 0.5 or 5 μg/mL CRP, 10 μM TRAP-6 and 5 μM ADP and binding of OG-Fg to activated platelets was measured in **B.** healthy donors (n=8), primary ITP patients (n=20) and control group (n=18). Primary ITP patients (n=3-16) are separated based on **C.** symptoms, **D.** time since diagnosis and **E.** first-line treatment (steroid and/or IVIg) at the time of sample collection. Data includes repeat measurement from the same subjects collected at different time points. A two-way ANOVA with Bonferroni’s multiple comparison test was performed.


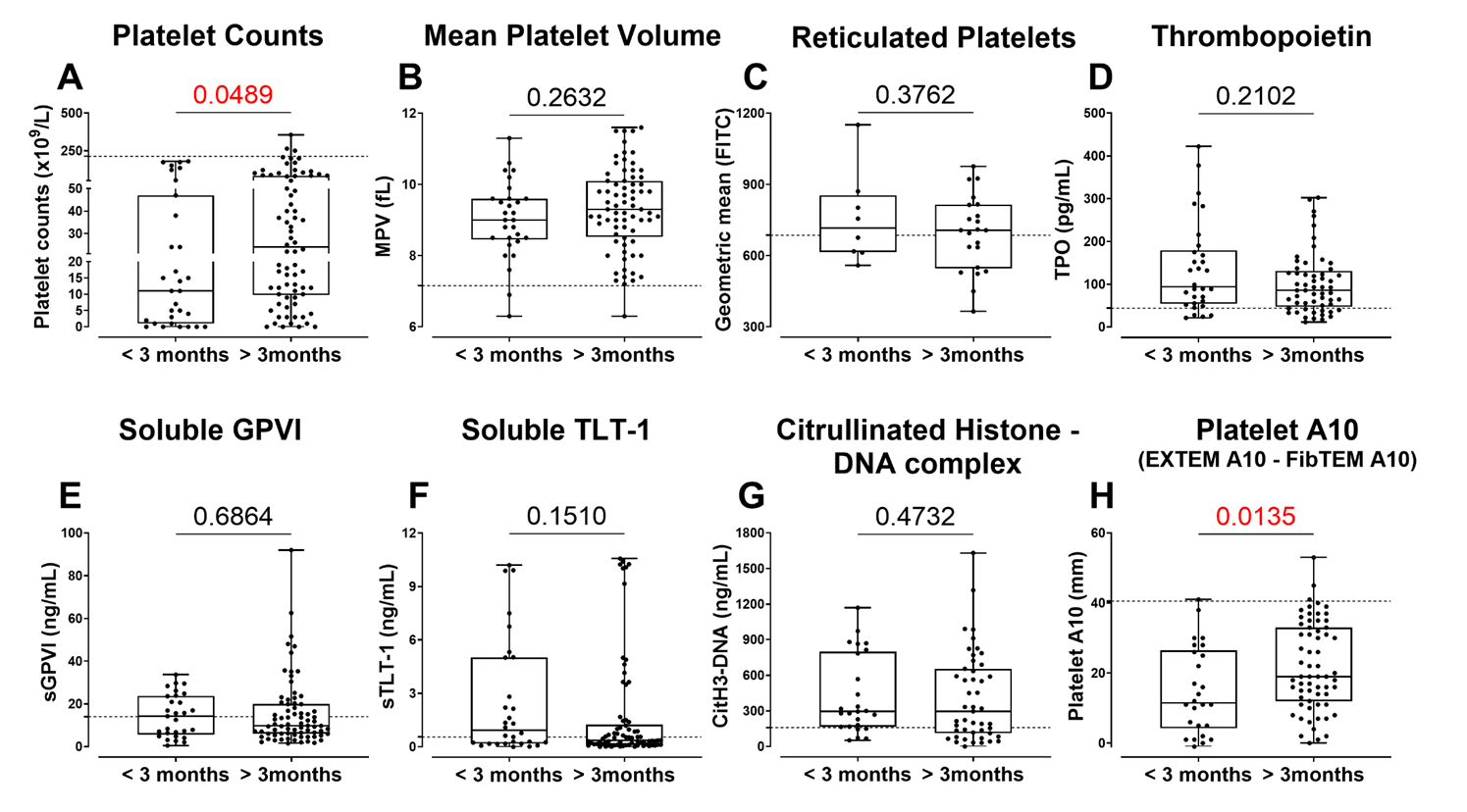


**Figure S3: Platelet and plasma parameters in primary ITP patients stratified for duration since diagnosis. A.** Platelet counts and **B.** MPV were measured using an automated haematology analyser in TSC-anticoagulated WB. **C.** Reticulated platelets were quantified by flow cytometry. Levels of **D.** TPO, **E.** sGPVI, **F.** sTLT-1 and **G.** CitH3-DNA complex were measured by ELISA. **H.** Platelet A10 was calculated by subtracting the FibTEM A10 measurement from the EXTEM A10 in ROTEM. The primary ITP patient cohort (n=8-75) includes repeat measurements from the same subjects collected at different time points. An unpaired t-test or Mann-Whitney test was performed depending on the distribution of the data. Significant p-values (p<0.05) are highlighted in red. The cutoff from HD levels is indicated by a dotted horizontal line on the respective graphs.


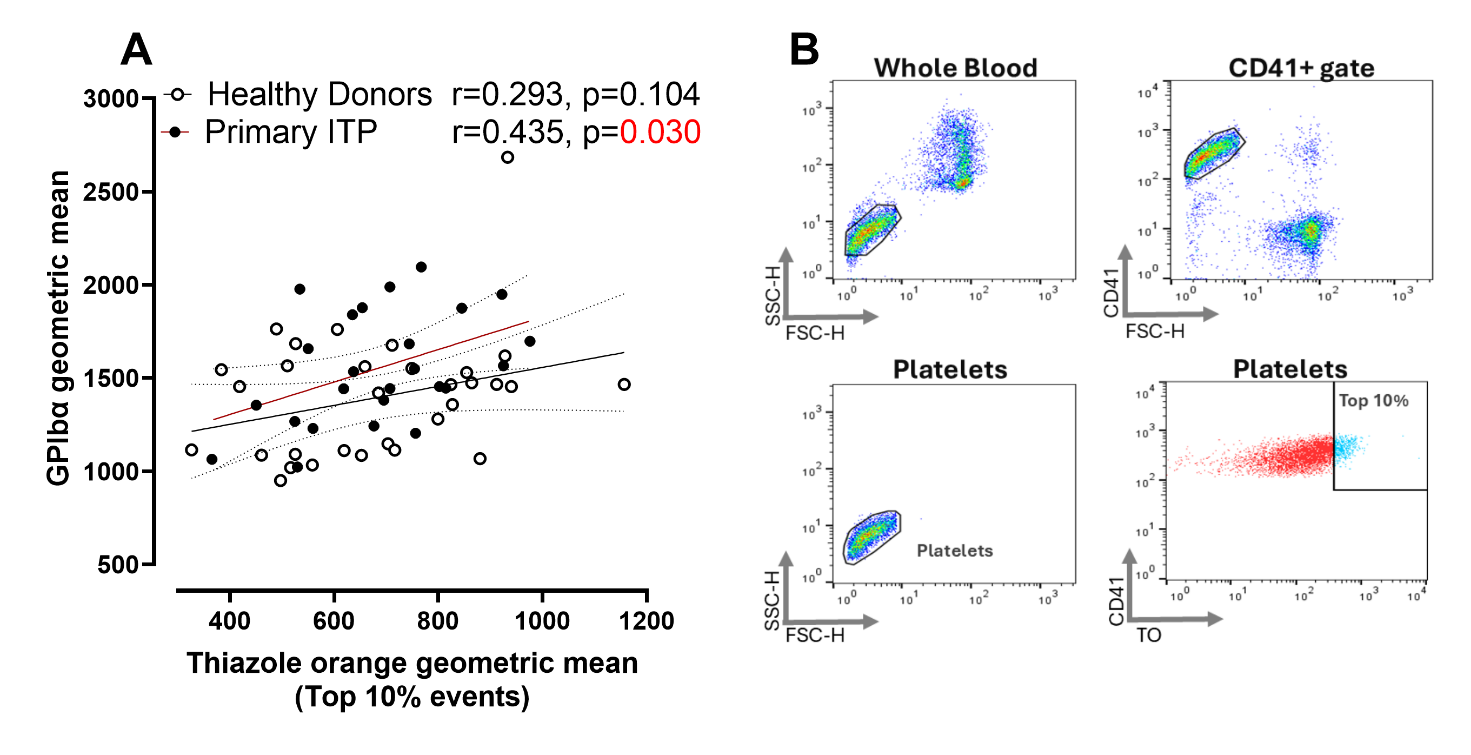


**Figure S4: Correlation between reticulated platelets and surface GPIbα levels.**

Reticulated platelets and surface GPIbα levels were quantified by flow cytometry using thiazole orange (TO) and a fluorescently conjugated antibody respectively. **A.** The correlation between reticulated platelet and GPIbα levels was calculated for healthy donors and (n=32) and primary ITP patients (n=25). **B.** Gating strategy for TO staining of platelets. The top 10% TO^bright^ events were gated within CD41^+^ gate. Significant p-value (p<0.05) is highlighted in red. Simple linear regression was performed with 95% confidence interval (dotted lines) and Pearson correlation coefficient (r) was calculated. FSC-H=forward scatter height, SSC-H=side scatter height, CD=cluster of differentiation, TO=thiazole orange.


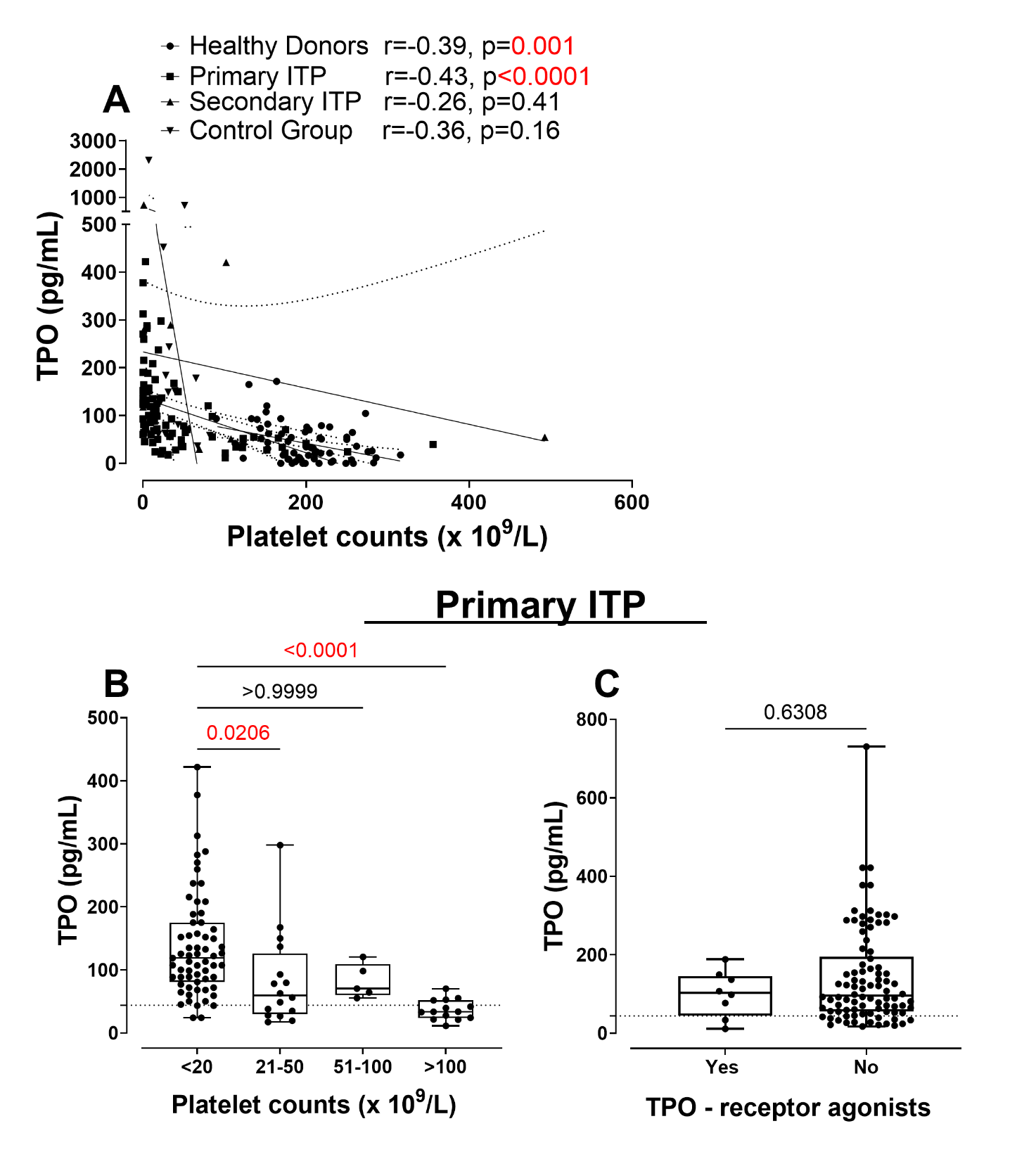


**Figure S5: Thrombopoietin levels in healthy donors, ITP and control group.**

Serum TPO was measured by ELISA. **A.** Correlation of TPO with platelet counts was calculated. TPO concentrations in primary ITP patients were then categorised based on their **B.** platelet counts and **C.** TPO-receptor agonist intake at the time of sample collection. Data (n=5-88) includes repeat measurements from the same subjects collected at different time points. Kruskal-Wallis with Dunn’s multiple comparison test (A, B) and a Mann-Whitney test (C) were performed between groups. Significant p-values (p<0.05) are highlighted in red. The cutoff from healthy donor serum TPO levels, measured alongside these patients, is indicated by a dotted horizontal line on the respective graphs.


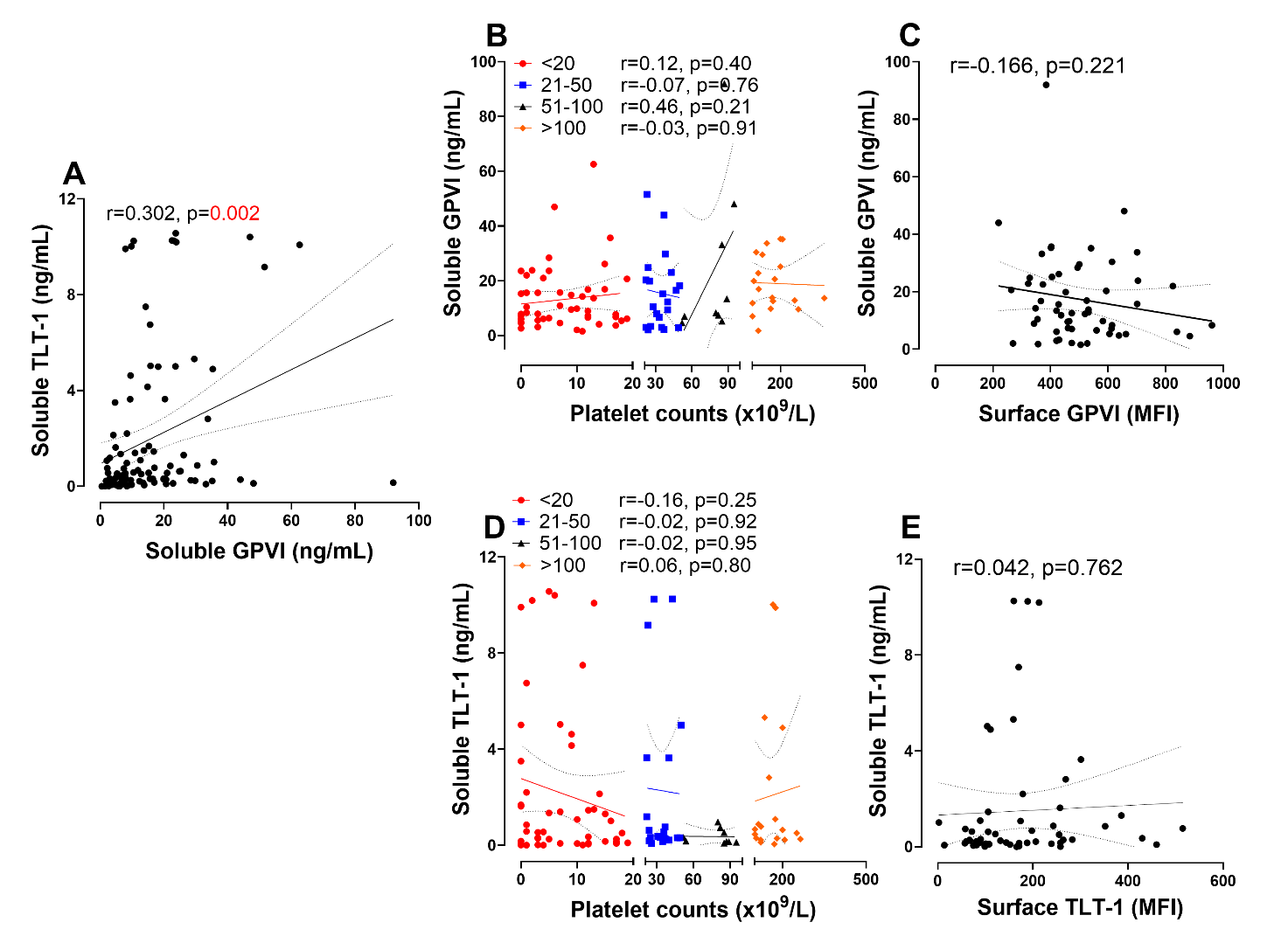


Figure S6: Correlation of soluble receptor levels with each other and platelet counts in primary ITP.

Soluble TLT-1 and soluble GPVI were measured in platelet-free plasma (n=9-105). **A.** Correlation between soluble TLT-1 and soluble GPVI in primary ITP patients. Correlation between **B.** soluble GPVI and **D.** soluble TLT-1 in primary ITP patients with platelet counts less than 20 x 10^9^/L, 21-50 x 10^9^/L, 51-100 x 10^9^/L and more than 100 x 10^9^/L. Correlation of C. soluble GPVI and **E.** sTLT-1 levels with their respective surface receptor levels. Significant p-value (p<0.05) is highlighted in red. Simple linear regression was performed with 95% confidence interval (dotted lines) and Pearson correlation coefficient (r) was calculated.


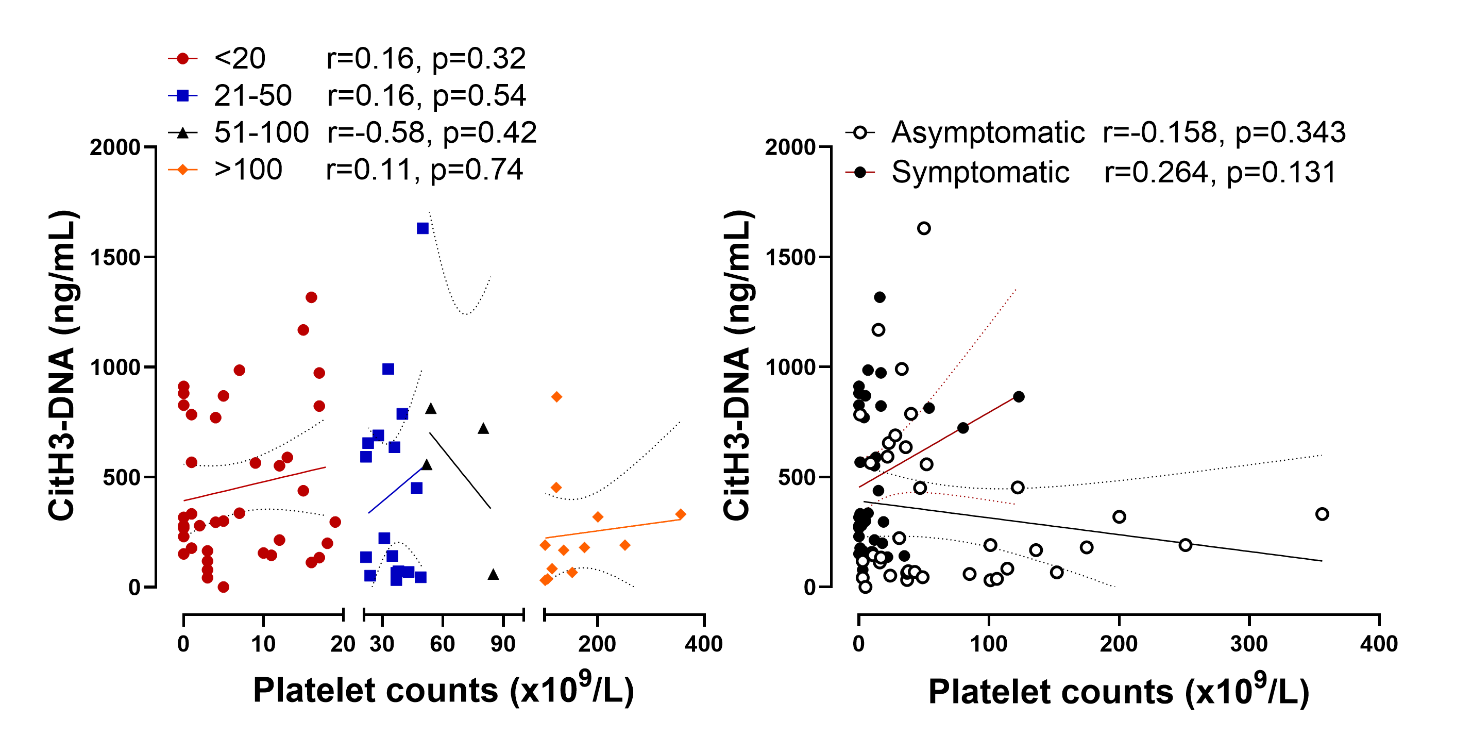
Figure S7: Correlation of CitH3-DNA complexes with platelet counts in primary ITP.

CitH3-DNA complexes were measured in platelet-free plasma from whole blood collected in cell-free DNA collection tube (white top) with (n=4-39). **A.** Correlation between CitH3-DNA and platelet counts in primary ITP patients with platelet counts less than 20 x 10^9^/L, 21-50 x 10^9^/L, 51-100 x 10^9^/L and more than 100 x 10^9^/L was calculated. **B.** Correlation between CitH3-DNA and platelet counts in ITP patients with (black circles) or without (open circles) symptoms of bleeding or bruising. Simple linear regression was performed with 95% confidence interval (dotted lines) and Pearson correlation coefficient (r) was calculated.


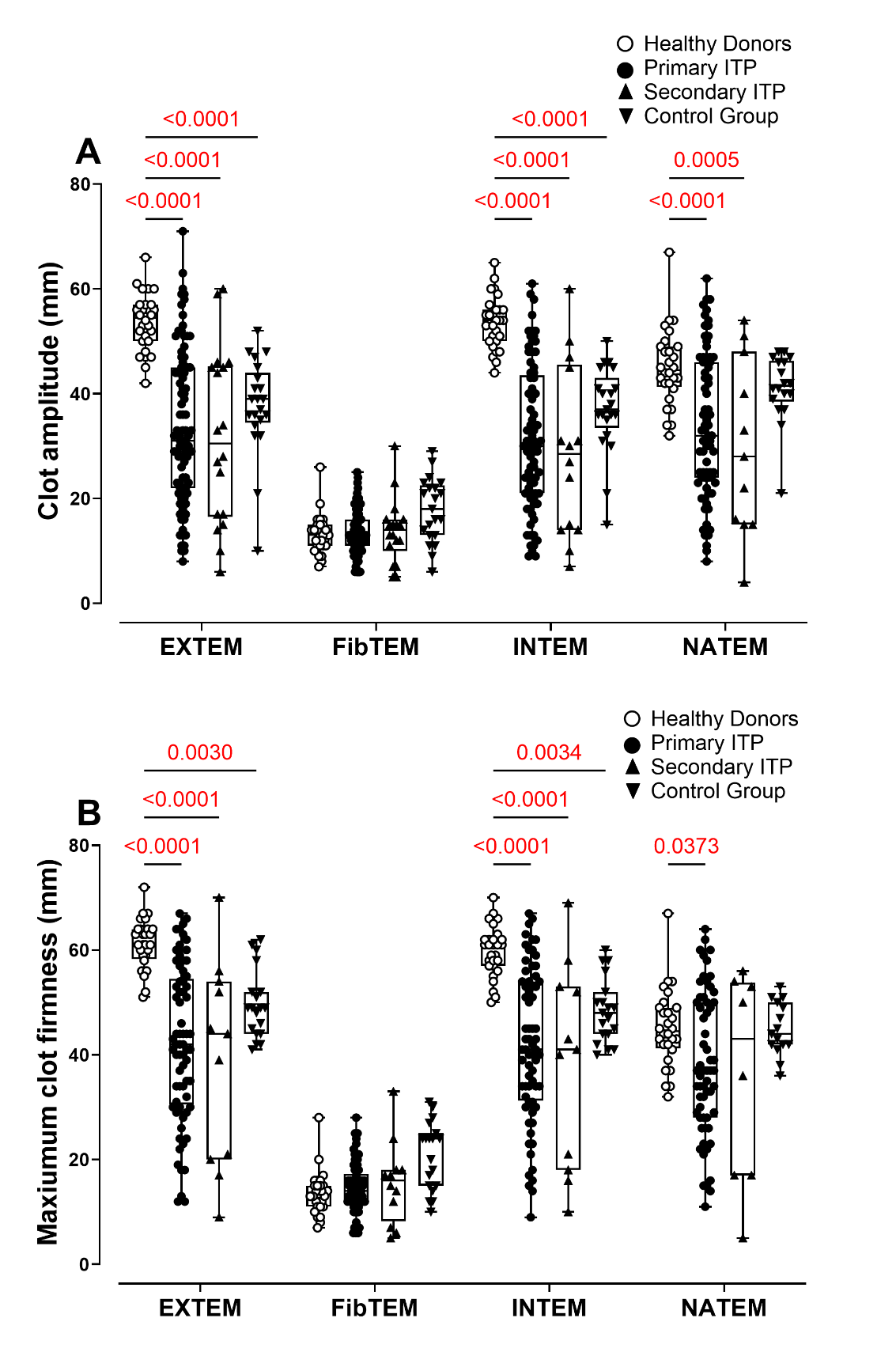


**Figure S8. ROTEM parameters in healthy donors, ITP patients and control group.** **A.** Clot amplitude at 10 minutes (mm) and **B.** maximum clot firmness (mm) were measured for EXTEM, INTEM, NATEM and FIBTEM in healthy donors (n=28), primary ITP (n=61-89), secondary ITP (n=8-18) and control group (n=15-21). A two-way ANOVA with Bonferroni’s multiple comparison test was performed. Only significant p-value (p<0.05) highlighted in red are displayed.


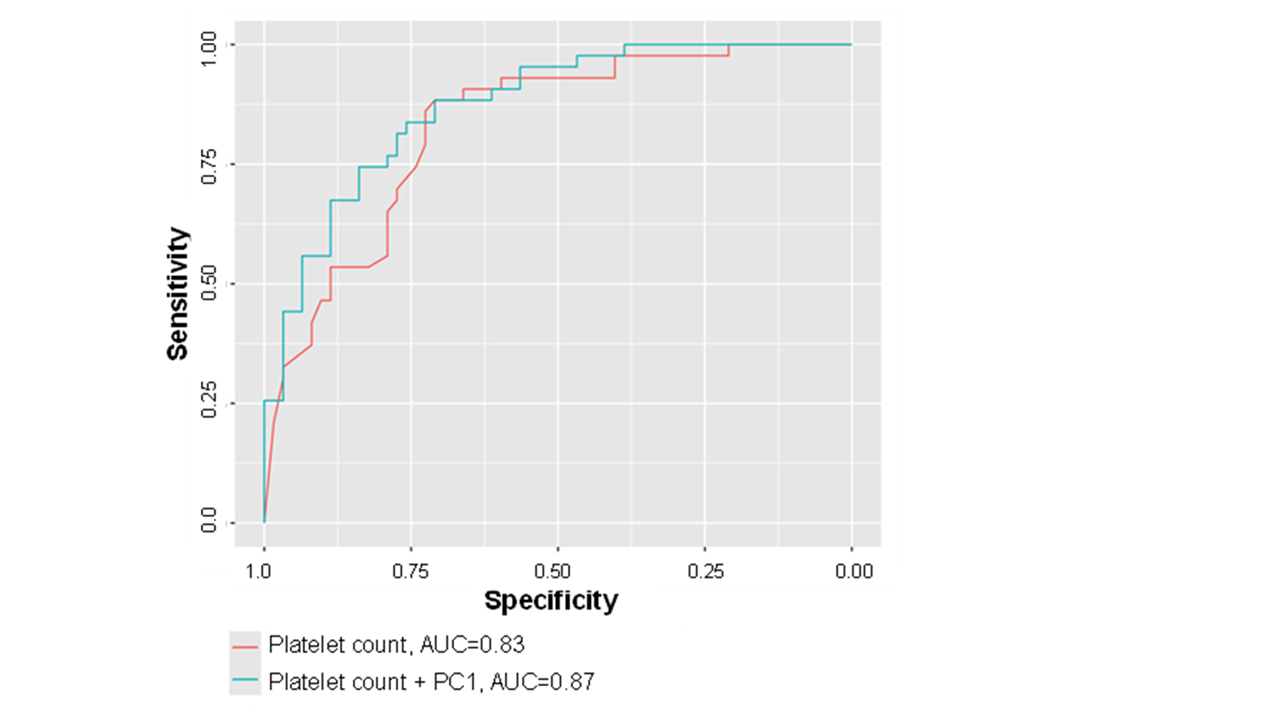


**Figure S9. Receiver Operating Characteristic (ROC) curves for risk prediction models.** The ROC curves compare the performance of two logistic regression models predicting the development of symptoms in primary ITP. Model 1 used platelet count as the sole predictor, while model 2 incorporated principal component 1 (PC1) alongside platelet count. The inclusion of PC1 improved model performance, reflected in a higher area under the curve (AUC), indicating enhanced predictive accuracy.
